# First-in-human quad-targeting CAR T cells for diffuse intrinsic pontine glioma: a phase 1 trial

**DOI:** 10.21203/rs.3.rs-11447687/v1

**Published:** 2026-10-27

**Authors:** Rebecca Ronsley, Marlena Bannick, Prabha Naraswayamy, Adam Beebe, Christopher T. Brown, Wenjun Huang, Stephanie D. Rawlings-Rhea, Jeffrey R. Whiteaker, Shrabanti Chowdhury, Andrea Timpanaro, Edward Z. Song, Leonel Elena-Sanchez, Michael Meechan, Audrey Alderman, Madison Beam, Tiffany Sad, Sam L. Bakotich, Matthew A. MacQuivey, Daniel V. Runco, Erin E. Crotty, Sarah E.S. Leary, Joelle P. Straehla, Juliane Gust, Alison Thomsen, Amy R. Beckstrom, Susan L. Holtzclaw, Corrine Hoeppner, Samuel R. Browd, Hannah E. Goldstein, Jason S. Hauptman, Amy Lee, Jeffrey G. Ojemann, Jason N. Wright, On Ho, Sowmya Pattabhi, Jason P. Wendler, Kamila S. Gwiazda, Pei Wang, Amanda G. Paulovich, Joshua A. Gustafson, Rebecca A. Gardner, Corinne Summers, Colleen Annesley, Julie R. Park, Michael C. Jensen, Nicholas A. Vitanza

**Affiliations:** 1 Ben Towne Center for Childhood Cancer and Blood Disorders Research, Seattle Children’s Research Institute, Seattle, WA, USA; 2 Department of Pediatrics, Seattle Children’s Hospital, University of Washington, Seattle, WA, USA; 3 Seattle Children’s Therapeutics, Seattle, WA, USA; 4 Fred Hutchinson Cancer Center, Seattle, WA, USA; 5 Department of Genetics and Genomic Sciences, Icahn School of Medicine at Mount Sinai, New York, NY, USA; 6 Norcliffe Foundation Center for Integrative Brain Research, Seattle Children’s Research Institute, Seattle, WA, USA; 7 Division of Pediatric Neurology, Department of Neurology, University of Washington, Seattle, WA, USA; 8 Division of Neurosurgery, Seattle Children’s Hospital & Department of Neurological Surgery, University of Washington, Seattle, WA, USA; 9 Barrow Neurological Institute at Phoenix Children’s Hospital, Phoenix, AZ, USA; 10 Department of Radiology, Seattle Children’s Hospital, Seattle, WA, USA; 11 Department of Pediatric Oncology, St Jude Children’s Research Hospital, Memphis, TN, USA

**Keywords:** CAR T cells, diffuse intrinsic pontine glioma (DIPG), diffuse midline glioma, H3K27-altered (DMG), intracerebroventricular delivery, multi-antigen immunotherapy

## Abstract

Diffuse intrinsic pontine glioma (DIPG) and diffuse midline glioma (DMG) are lethal pediatric central nervous system (CNS) tumors with a median survival of 11 months. Despite advancements in translating chimeric antigen receptor (CAR) T cells for these patients, efficacy may be constrained by antigen heterogeneity and immune escape. We conducted a first-in-human, phase 1 trial (BrainChild-04; NCT05768880) evaluating repetitive intracerebroventricular (ICV) administration of a multi-antigen targeting CAR T cell product directed against B7-H3, EGFR806, HER2 and IL13Rα2 (quad-CAR T cells) for children and young adults with DIPG/pontine DMG (Arm A) and recurrent or refractory CNS tumors (Arm B). Here we report results from Arm A. Primary endpoints were feasibility, safety, and determination of the maximally tolerated dose regimen (MTDR). Secondary endpoints included CAR T cell distribution and preliminary clinical activity. Correlative analyses assessed CAR T cell kinetics and cytokine dynamics in cerebrospinal fluid (CSF). Twenty-six patients were enrolled with 100% manufacturing success rate. Twenty-one patients received at least one infusion (median 8/patient, range 3-56). No dose-limiting toxicities occurred in the dose-finding period, and treatment was tolerable with predominantly grade 1-2 adverse events, including fever (95%), headache (86%), and fatigue (81%). One dose-limiting toxicity (acoustic nerve disorder) occurred outside the evaluation window. Repetitive ICV dosing was feasible, and most of the infused patients (86%) initiated two or more treatment courses (median 4). CAR T cells were detected in CSF in 100% of patients, across serial courses in 76% of patients, with evidence of detection across sequential courses, particularly at higher dose levels. Cytokine profiling demonstrated dynamic immune activation within the CNS. Median survival from initial CAR T infusion was 8.6 months (95% CI: 5.2-10.3). All deaths were due to disease progression. Repetitive ICV administration of quad-CAR T cells is feasible and tolerable in children and young adults with DIPG/DMG. This study represents the first clinical application of quad-antigen targeting CAR T cells and confirmed feasibility in manufacturing and preliminary evidence of biologic activity in the CNS. These findings support further evaluation of multi-antigen CAR T strategies to address tumor heterogeneity of antigen expression as a likely barrier to CAR T cell efficacy in patients with CNS tumors.

## INTRODUCTION

Tumors of the central nervous system (CNS) are the most common solid malignancy in children and young adults and remain the leading cause of pediatric cancer-related mortality.^1,2^ Diffuse intrinsic pontine glioma (DIPG) and diffuse midline glioma, H3K27-altered (DMG) account for approximately 10% of pediatric CNS tumors and carry a dismal prognosis, with median survival of approximately 11 months.^3,4^ Recent advances in molecular profiling have improved biologic understanding and risk stratification of these tumors, but molecular targeting infrequently leads to durable responses.^5^ Many other therapies ranging from conventional chemotherapies to novel therapeutics remain constrained by poor blood brain barrier penetration, tumor heterogeneity, immunosuppressive microenvironments, and adaptive resistance mechanisms.^6–9^

Chimeric antigen receptor (CAR) T cell therapy has emerged as a promising strategy for pediatric CNS tumors. CAR T cells mediate major histocompatibility complex independent recognition of tumor associated antigens and may be particularly suited to CNS malignancies through locoregional delivery into the cerebroventricular system or tumor cavity, enabling high local effector concentrations with limited systemic exposure.^10–13^ Early clinical studies targeting B7-H3, EGFR806, GD2, HER2, or IL-13Rα2, demonstrated the feasibility and relative safety of intravenous (IV) or intracerebroventricular (ICV) CAR T cell administration to children and adults with CNS tumors.^14–20^ However, antigen heterogeneity and immune escape through antigen loss or downregulation remains a major challenge for CAR T cells against solid tumors.^21^ In particular, pediatric high grade glioma (HGG) exhibit spatially and temporally variable antigen expression, suggesting that multi-antigen targeting may lead to more durable tumor control.^21^ Multi-antigen CAR T cell strategies have therefore emerged as a potential approach to broaden tumor coverage and reduce immune escape, although clinical experience in pediatric CNS tumors remains limited.

Building on prior experience with single antigen targeting CAR T cell clinical trials using repetitive ICV delivery for children and young adults with CNS tumors, our experience with repetitive ICV B7-H3 CAR T cells demonstrated the feasibility and tolerability of prolonged locoregional CAR T cell therapy in children and young adults with DIPG, with evidence of CAR T cell detection in the CSF, local immune activation, and potential clinical benefit.^14–16^ Next, to assess the feasibility and tolerability or multi-antigen targeting we opened BrainChild-04 (NCT05768880), evaluating repeatedly dosed CAR T cells targeting B7-H3, EGFR, HER2, and IL-13ra2 (quad-CAR T cells). Here, we report outcomes from BrainChild-04’s Arm A, which enrolled children and young adults with DIPG/pontine DMG.

## METHODS

### Preclinical in vitro studies

U87-MG (ATCC) cells were transduced and purified to express the human HER2 antigen, GFP, and firefly luciferase. Cells were individually stained for antigens B7-H3 (anti-human CD276-APC, Biolegend, 351006), EGFR (anti-human EGFR-APC, Biolegend,Cat 392506), HER2 (Herceptin-APC, BD, 624367), and IL13Ra2 (anti-human CD213a2-APC, Biolegend, 354406). Antigen positivity was determined by gating compared to unstained control. A BD LSRII flow cytometer (BD Biosciences, San Jose CA) was used to acquire samples and data was analyzed using FlowJo (BD Life Sciences) software.

Mock and quad-CAR T cells were thawed, expanded for two weeks using a Rapid Expansion Protocol^22^ and then harvested for an Incucyte Cytotoxicity Assay (Sartorius, Gottingen, Germany). Cells were counted, washed, and seeded in triplicate at 5:1 CAR T cell to target ratio in a 96-well flat bottom plate, at a final concentration of 50,000 CAR T cells/well. Target cell cultures were counted, washed, and then seeded into the same 96-well flat bottom plate at a final concentration of 10,000 target cells/well. The 96-well flat bottom plates containing both target and T cells was incubated at room temperature for 30 minutes and then placed in the IncuCyte S3 imager. Live-cell images were taken every 3 hours for the duration of the assay and analyzed using the IncuCyte Base Analysis Software (Version 2022, Sartorius). The primary models used for in vitro luciferase assays were modified to express firefly luciferase. D-Luciferin (Revvity, #122799-5) substrate solution was added to the cell culture at the 48-hour time point and incubated at room temperature for 15 min before quantifying the bioluminescent signal from each well with a SpectraMax iD5 (Molecular Devices).

### Preclinical in vivo studies

All mouse experiments were conducted in accordance with the IACUC-approved protocols ACUC00736 and ACUC00669 at Seattle Children’s Research Institute. For the orthotopic intracranial xenograft model^23^ with U87-MG HER2+, male eleven- to twelve-week-old NOD/*Scid* IL-2RgC^null^ (NSG) mice were intracranially injected on day 0 with 200,000 U87 HER2^+^ GFP+ luciferase+ tumor cells in 2 uL PBS (Gibco, Thermo Fischer Scientific, Waltham MA) using a stereotactic surgical setup with the coordinates of 2 mm lateral and 0.5 mm anterior to bregma, and 2.5 mm deep from dura. Seven days after tumor implantation, 2 x 10^6^ CAR T cells in 3 μL PBS were injected 2.5, 2.35, and 2.25 mm deep from the dura. To determine the background bioluminescent signal without tumor, mice were imaged on day −1 using the IVIS Spectrum Imaging System (Revvity, Waltham MA). Imaging of mice took place on day 6 to establish tumor engraftment prior to T cell product administration. Mice were subsequently imaged regularly until day 84 to track tumor progression. All images were analyzed using the Living Image software (Perkin Elmer, Waltham MA) for bioluminescent signal represented as total flux (photons/second). Death was defined as euthanasia due to symptoms related to tumor progression.Survival was analyzed using Kaplan Meier methods. For the orthotopic xenograft model with PBT-27FH, gender-balanced nine-week-old NSG mice (Jackson Laboratories) were intracranially injected with 350,000 PBT-27FH cells in 2 μL PBS (Cytiva, #SH30256.LS) using a stereotactic surgical setup with the coordinates of 0.8 mm lateral and 1.5 mm posterior from lambda, and 2.5 mm deep. Seven days after tumor implantation, 2 x 10^6^ CAR T cells in 3 μL PBS were injected 2.5, 2.35, and 2.25 mm deep from the dura. Mice were anesthetized by intraperitoneal injected cocktail of ketamine (Patterson Veterinary Supply, #07-894-8462; 100 mg/kg animal) and xylazine (Patterson Veterinary Supply, #07-808-1947; 8 mg/kg animal) for the surgeries and given analgesic buprenorphine (Wedgewood Connect, #BUPREN-INJ010VC; 1 mg/kg animal, extended release) by the veterinary staff before the surgeries. Murine weights were recorded on a weekly basis with routine health checks to monitor any sign of illness such as weight loss, hair loss, cranial distension, lethargy, and hunching posture at rest. Mice with severe symptoms were euthanized based on protocol-defined criteria.

### CAR T cell Product Manufacturing

The investigational quad-CAR T cell product is manufactured from autologous peripheral blood mononuclear cells (PBMC) which are obtained from the patient via standard leukapheresis collection procedures. The fresh apheresis products are delivered to a Good Manufacturing Practice (GMP) facility for further processing. PBMCs undergo simultaneous positive selection for CD4^+^ and CD8^+^ T cells using the CliniMACS Plus System (Miltenyi). The T cells are seeded into culture at a prescribed cell number, activated with GMP-grade CD3/CD28 CTS Dynabeads (Thermo) and then simultaneously transduced with varying individual vector volumes sufficient to achieve effective efficiency and copy number with a mixture of four third-generation HIV7-based self-inactivating lentiviral vectors, each encoding one of the four CARs. Both the B7-H3CAR^24^ and the IL13zetakine^25^ constructs additionally encode a truncated EGFR (EGFRt) transduction marker^26^ and the selection transgene dihydrofolate reductase double mutant (DHFRdm),^26^ which allows for in vitro selection using methotrexate (MTX). Both the EGFR806^14^ and HER2^15^ constructs co-express EGFRt as well, but without the DHFRdm transgene. The stimulated T cells were expanded in the presence of methotrexate to enrich for cells transduced with B7-H3 and/or IL-13Rα2 CARs over an approximately 7-14 days period ex vivo and then harvested to obtain the necessary number of EGFRt^+^ cells needed for a therapeutic dose. The inclusion of DHFRdm and MTX selection was an evolved component of the vector/manufacturing platform that was not in place when first two vectors were manufactured. Cell products were washed and formulated in Cryostor CS5 cryopreservation media and filled into an appropriate number of CellSeal cryovials (Sexton), with each vial containing sufficient cryopreserved cells for patient administration. After cryopreservation, the patient specific quad CAR T cell product underwent release testing (assays included: viability, endotoxin, mycoplasma, sterility, immunophenotyping, vector copy number, residual bead assay, HLA typing, vector confirmation, and residual methotrexate). The cryopreserved product was stored in the vapor phase of liquid nitrogen prior to shipment to the investigator’s site. Following receipt, the cell product was diluted into Normosol-R to prepare it for patient administration.

### Trial Objectives

The primary aims of this trial were to assess the feasibility, safety, and tolerability; and to describe the maximally tolerated dose regimen (MTDR) of repeatedly dosed ICV quad-CAR T cell therapy for children and young adults with DIPG/pontine DMG (Arm A) and recurrent or refractory CNS tumors (Arm B). Documentation of tumor antigen expression was not required. Feasibility is defined as (1) number and percent of patients with sufficient therapeutic product generated to receive two courses at the intended dose level (DL) per assigned dose regimen (DR) after two attempts using a single apheresis product for starting material, (2) number of patients who meet criteria for initial CAR T cell infusion, and (3) number of patients who meet criteria for at least two courses of CAR T cell infusions. Safety and tolerability were determined by adverse events as assessed by the Common Terminology Criteria for Adverse Events (CTCAE v5.0). Adverse events were recorded from initial CAR T cell infusion through 28 days after a patient’s final infusion. A DLT was defined as any ≥ grade 3 AEs deemed related to the CAR T cell product with the following exceptions: grade 3 cytokine release syndrome (CRS) that decreases to ≤ grade 2 within 72 hours; ≥ grade 3 hypotension, fever, and/or chills not controlled with medical intervention that decreases to ≤ grade 2 within 72 hours; ≥ grade 3 activated PTT, fibrinogen, and/or INR that are asymptomatic and resolve within 72 hours; ≥ grade 3 hypoglycemia and/or electrolyte imbalance that are asymptomatic and resolve within 72 hours; ≥ grade 3 nausea and/or vomiting that decrease to ≤ grade 2 with 7 days; and grade 3 neurologic symptoms that decreases to ≤ grade 2 within 21 days. The definition of a DLT also included any toxicity lasting > 14 days that prevents the patient from meeting criteria for subsequent CAR T cell infusion in Courses 1 or 2. Since some patients may receive infusions indefinitely, the DLTs used for dose escalation decision making and MTDR determination were time-restricted (see Statistical Analysis for details).

Secondary objectives for this trial were (1) to assess quad CAR T cell distribution within the cerebrospinal fluid (CSF) and the extent to which quad CAR T cells egress into the peripheral circulation and (2) to assess disease response following fractionated intraventricular CNS administration of quad-CAR T cells in children and young adults with DIPG or pontine DMG. The trial radiologic response criteria assessed by magnetic resonance imaging (MRI) after Course 1 and every two subsequent courses utilized the standard sum of the two longest 2D perpendicular diameters to distinguish: stable disease, progressive disease (>25% increase), partial response (>50% decrease), and complete response (no evaluable or measurable disease).^27^

### Tumor Inflammation Associated Neurotoxicity

Although tumor inflammation-associated neurotoxicity (TIAN)^28,29^ grading had been published, it was not permitted as a mechanism to classify adverse events at the time of this trial; therefore, prospective assessment and documentation of TIAN was not done. To evaluate the potential presence of TIAN retrospectively, review of study data and patient medical records was performed. Clinical symptoms, radiographic findings, and treatment context were assessed to determine whether individual cases met published TIAN criteria.^28^

### Patients

DIPG was defined using RAPNO MRI criteria including tumors with expansion of the pons and T2/FLAIR hyperintensity involving at least 50% of the pons, with ill-defined margins and variable contrast enhancement and without dominant exophytic component.^30^ DMG was defined using the World Health Organization (WHO) 2021 criteria^31^. Enrollment criteria for Arm A included: age ≥ 1 and ≤ 26 years evidence of DIPG on MRI or biopsy-proven DMG with the primary lesion within the pons at any timepoint following completion of standard radiation; ability to tolerate apheresis; presence of a CNS catheter;^32^ life expectancy ≥ 8 weeks; Lansky/Karnofsky performance of ≥ 60; defined washout periods from prior therapies; adequate organ function including absolute lymphocyte count (ALC) ≥ 100 cells/μL, absolute neutrophil count ≥ 500 cells/μL, hemoglobin ≥ 9 g/dL, platelets ≥ 100,000/μL, creatinine ≤ upper limit of normal (ULN) for age, total bilirubin < 3x ULN for age or conjugated bilirubin < 2 mg/dL, an oxygen saturation ≥ 90% on room air without dyspnea at rest, adequate neurologic function defined as stable deficits for ≥ 1 week, ≤ 2 anti-epileptic agents required to control seizures, and no encephalopathy; negative virology for HIV, Hepatitis B, and Hepatitis C; and use of highly effective contraception in patients of child-bearing age^33^. Exclusion criteria included: severe cardiac dysfunction; primary immunodeficiency or bone marrow failure syndrome; evidence of impending CNS herniation; another active malignancy; severe, active infection; active receipt of any anti-cancer therapy; or pregnancy or breastfeeding.

### Trial Design

This was a phase 1 study of CNS ICV adoptive quad-CAR T cell therapy, delivered by an indwelling catheter in the ventricular system in children and young adults with radiographic DIPG or biopsy confirmed pontine DMG (Arm A) (BrainChild-04; NCT05768880). BrainChild-04 began accrual on May 5, 2023, with the first patient enrolled on Arm A on October 23, 2023, and last patient enrolled on Arm A on October 1, 2025. Data collected through April 15, 2026 is included in this manuscript. This study was conducted in accordance with US Food and Drug Administration and International Conference on Harmonization Guidelines for Good Clinical Practice, the Declaration of Helsinki, and approved by the Seattle Children’s Institutional Review Board. The study was monitored by a data monitoring committee. Enrolled patients underwent leukapheresis. CD4+ and CD8+ T cells were selected from apheresis products and subsequently bioengineered to create the investigational product. BrainChild-04 Arm A patients receive ICV infusions through a CNS catheter on Weeks (W) 1, 2 and 3 of each 4-week Course (Cr), with intra-patient dose level (DL) escalations in all dose regimens (Figure 2). Lymphodepletion was not incorporated^34,35^. Patients enrolled into one of the three escalating dose regimens (DRs). All DR employed intra-patient dose escalation.

Requirements to receive a CAR T infusion included: a CNS catheter in place ≥ 5 days from surgery; evidence of persistent, evaluable disease; not breastfeeding nor pregnant; meeting defined washout periods from any bridging therapy; adequate organ function defined by specified laboratory values used for eligibility; no encephalopathy or uncontrolled seizure activity; compliance with prescribed anti-epileptic drug administration; and no evidence of active severe infection. To receive subsequent infusions, patients were required to have had no DLT after prior infusions. After Course (Cr) 1, patients could receive continued doses for an unlimited number of courses if meeting ongoing dosing eligibility criteria and received at least one dose every five weeks. Response was continually assessed; following Course 1 and subsequently after even numbered courses, using MRI brain and spine, and CSF cytology. Overall response accounts for target lesions, non-target lesions, and new lesions according to the criteria defined in Supplementary Table 4, including: complete response (CR), partial response (PR), stable disease (SD), and progressive disease (PD). A patient’s best overall response is the best recorded overall response that is seen from their initial infusion until either the first recorded overall response of PD, or treatment discontinuation (whichever occurs first). Follow-up is ongoing, with patients monitored until death or for 15 years in long-term follow-up.

### Statistical Analysis

All patients who received at least one CAR T cell infusion were included in safety and efficacy analyses. Baseline demographic and clinical characteristics were summarized using descriptive statistics. Adverse events were reported systematically throughout the MTDR Evaluation Period. Patients who died from toxicity before the first response assessment were considered non-responders.

All survival analyses only include infused patients. Survival was calculated as the time from initial infusion to death from any cause. To enable comparisons with historical data and other published cohorts, survival from time of diagnosis was also calculated. However, it is a quantity that carries immortal time bias since it represents a distinct cohort of diagnosed patients who lived long enough to enroll in the study and receive an initial infusion. To aid in the interpretation of this quantity, we also report the median time from diagnosis to enrollment among both enrolled and infused patients, and median time from enrollment to initial infusion among infused patients. Patients were censored at the last study contact, or the date of withdrawal of consent. Survival was analyzed using the Kaplan-Meier estimator. Pointwise 95% confidence intervals for the survival functions were constructed using Greenwood’s variance estimate with the complementary log-log transformation. Confidence intervals for median survival were calculated using the Brookmeyer-Crowley method. If the upper limit of the 95% CI for the median is not reached, it is denoted with “NR”.

For MSD analysis, differential expression between pre- and post-infusion timepoint was assessed using mixed linear-effects models (lme4 package 2.0-1),^36^ with study participants included as random intercepts and log2-transformed cytokine expression values as the response variable. A single model was fitted to include all 47 cytokines and 1 paired timepoint during the DLT period, and linear contrasts, calculated using estimated marginal means (emmeans package 2.0.3)^37^ were calculated to evaluate pre- versus post-infusion differences at the timepoint.

For the targeted PRM data analysis, preprocessing was performed that included the following steps. First, the missing peak area ratio (PAR) values were imputed by one-third of the limit of quantitation (LOQ). Next, PAR values were normalized by the input volume; and the normalized imputed values per peptide were replaced by the minimum of the imputed values. Finally, log transformation was applied on the normalized PAR. Statistical analyses were conducted to study protein expression changes after treatment at different time points compared to baseline pretreatment CSF samples. Specifically, a linear mixed effect regression analysis was performed to jointly study the effect of treatment after time points Cr1W2 (considering both pre and post treatment samples) and Cr1W3 (pre-treatment samples) and considering a random effect to account for the patient level variability: *Protein expression ~ Cr1W2_pre + Cr1W2_post + Cr1W3_pre + random (1| patient)*. A trend analysis was also performed using a linear mixed effect model considering the pre-treatment samples of the time-points: Cr1W1, Cr1W2, Cr1W3, Cr2W1, Cr3W1, Cr4W1, Cr5W1 and Cr6W1, and treating the time points as ordinal. *Protein expression ~ time-points + random (1| patient)*, where time-point variable was coded as numeric 1 to 8 for the 8 time-points (in the order above), respectively.

Analyses were conducted in SAS version 9.4 and R version 4.5.1.

### Sample Collection and Processing

Cerebrospinal fluid (CSF) samples were obtained from patients via lumbar puncture or ventricular catheter and maintained at 4°C until same day processing. The samples were centrifuged at 250xg for 10 minutes to collect the cellular portion for immunophenotyping analysis. The supernatant was further spun at 10,000xg for 10 minutes to eliminate any remaining debris. The resulting cell-free CSF supernatant was aliquoted and cryopreserved for subsequent cytokine and chemokine analysis. The cellular fraction of the CSF was maintained at 4°C in AIM V Medium (ThermoFisher) before immunophenotyping analysis.

### Immunophenotyping and CAR Detection in CSF and Peripheral Blood

Peripheral blood collected in EDTA-treated tubes was stored at room temperature before treatment with red blood cell lysis buffer (eBioscience). Lysed peripheral blood and CSF cells underwent immunophenotyping analysis using two versions of a standardized CAR detection panel with fluorophore-conjugated antibodies (Supplementary Table 1). Cells were acquired on an LSRFortessa (BD Biosciences), and flow cytometric analysis was performed using FlowJo software (BD Biosciences). CAR detection was performed using a standardized gating strategy (Supplemental Figure 1A). CAR T cells were defined as singlets/viable/cells of interest (non-red blood cells or debris)/lymphocytes/CD3+/EGFRt+ (transduction tag). To ensure specificity, background noise from a fluorescence-minus-one (FMO) control was subtracted from the fully stained sample to determine total CAR-positive events. Samples with lymphocyte counts below the limit of quantitation (LoQ) of 200 lymphocytes for the assay were excluded from further analysis. CAR T cell detection status was confirmed when the percentage of EGFRt+ cells within the lymphocyte population exceeded the pre-defined LoD, 0.169% for V00 and 0.04% for V01, for the assay. Co-expression of activation marker expression was analyzed via Boolean gating.

### Direct CAR detection in Drug Products

Immunophenotyping and specific CAR expression profiling of cryopreserved drug products was performed following thaw using standard staining and flow cytometry techniques using fluorophore-conjugated anti-human monoclonal antibodies or peptides specific to each CAR (**Supplemental Table 2**). CAR T cell expression was quantified using an anti-human monoclonal antibody for EGFRt and IL-13, and recombinant human proteins B7-H3, HER2/ERBB2 Protein, and EGFRvIII. Stained samples were acquired on a Cytek Aurora (Cytek Bioscience) and flow cytometric analysis was performed using FlowJo software (BD Biosciences). CAR T cells were defined as singlets/viable/lymphocytes/CD3+/EGFRt+ (Supplemental Figure 1B). Co-expression of the four CAR constructs was analyzed via Boolean gating.

### Electrochemiluminescence Assay using Meso-Scale Discovery (MSD)

Patient cerebral spinal fluid (CSF) samples were thawed and analyzed for 47 analytes per manufacturer specifications with methodology previously published.^16^ In brief, this was done using the following Meso-Scale Discovery (MSD) V-PLEX plus human biomarker kits: V-PLEX Plus Angiogenesis Panel 1 (human) Kit (MSD, Cat. No. K15190G), V-PLEX Plus Chemokine Panel 1 (human) Gen. B Kit (MSD, Cat. No. K15705D), V-PLEX Plus Cytokine Panel 1 (human) Kit (MSD Cat. No. K15050D), V-PLEX Plus Cytokine Panel 2 (human) Kit (MSD, Cat. No. K15084D), V-PLEX Proinflammatory Panel 1 (human) Kit (MSD, Cat. No. K15049D), and V-PLEX PLUS Vascular Injury Panel 2 (human) Kit (MSD, Cat. No. K15198D. All CSF samples were diluted per manufacturer’s recommendations apart from analytes IP10 and MCP-1 of the V-PLEX Plus Chemokine Panel 1 (human) Gen. B Kit, which were diluted 50-fold to prevent signal saturation. Samples were all plated in duplicates. Experiment set up was performed and carried out per manufacturer’s protocol. Samples were read on the MESO QuickPlex SQ 120 instrument using the MSD discovery Workbench 4.0.12 software. Standard curves were first established from the standards using a five-parameter logistic model fitting algorithm developed in-house in R. Analyte concentrations were then determined by extrapolation from respective standard curves. To ensure data integrity, cytokine concentration above the upper limit of detection (ULoD) were assigned to the corresponding ULoD values.

### Proteomics Analysis - Sample processing

CSF specimens were processed in random order using 200 μL aliquots, where available, per timepoint. A mixture of cleavable stable isotope labelled standards was added at 50 fmol for each analyte and 50 μL of 20% Deoxycholate (Fisher Scientific B349)/0.03M TCEP in 0.19M Tris (pH 10.8) was added and incubated at 40°C for 1 hour. The samples were removed from the thermomixer and alkylated with 50 μL 0.06M Iodoacetamide for 30 minutes in the dark at room temperature. Samples were diluted with 100 μL Tris (pH 10.8) and 50 μL of trypsin (10 mg/mL in Tris, pH 10.8; Promega sequencing grade) was added and allowed to incubate for 4 hours at 37°C. Following digestion, 10 μL TLCK (3 mg/mL in 10mM HCl; Sigma Aldrich T4661) was gently added with shaking for 5 minutes.

Enrichment was performed as previously described^15^ with the following modifications. Antibodies were crosslinked on protein G magnetic beads (GE Sepharose) and peptide enrichment was performed by adding 1 μg antibody per target to the digested CSF. Following overnight incubation, beads were washed twice in 1X PBS buffer + 0.01% CHAPS, washed once in 1/10X PBS + 0.01% CHAPS, and peptides were eluted in 100 μL of 5% acetic acid/3% acetonitrile/50 mM citrate using a KingFisher automated processor (Thermo Fisher Scientific). The eluted peptides were loaded on Evotips (Evosep Biosystems) for analysis.

### Proteomics Analysis - Liquid Chromatography-Tandem Mass Spectrometry (LC–MS/MS)

Targeted LC–MS/MS was performed using an Orbitrap Astral platform (Thermo Fisher Scientific) with an Evosep One (Evosep Biosystems) LC system configured for low-flow peptide separations (“Zoom” methods). Peptides were separated by a 40 samples per day reversed-phase chromatography method on an Aurora 8cm x 75μm C18 column (IonOpticks) interfaced to the mass spectrometer using an EasySpray source (Thermo Fisher Scientific). Targeted MS/MS spectra (i.e., Parallel Reaction Monitoring, PRM) were collected using 100% AGC and maximum fill time of 2 ms. Retention time scheduling was optimized empirically and set to 2-minute windows. Endogenous (“light”) peptides were quantified relative to spiked heavy stable isotope–labelled peptides. Raw data were processed in Skyline^38^, with peak boundaries reviewed manually. Peptide identity was confirmed by retention time alignment and concordance of fragment ion patterns between light and corresponding heavy peptides. Transitions/fragments exhibiting interference were excluded. Quantification was performed using summed peak areas for each light/heavy pair, and light/heavy ratios were calculated per peptide. Measurements were retained only if they exceeded the lower limit of quantification (LLOQ/LOQ) as defined from prior analytical characterization.^39–43^

### Dose Regimen-Finding Design and MTDR Determination

Dose regimen escalation followed a modified Bayesian Optimal Interval Design (BOIN)^44^ with a target toxicity rate of 30%. Only DLTs occurring during the *Dose Regimen (DR) Escalation Evaluation Period*, defined as the time from the first infusion until 7 days post-final infusion in Cr1, guided dose escalation decisions in the BOIN design. Patients who discontinued protocol therapy without a DLT before the end of the DR Escalation Evaluation Period were deemed not evaluable for dose escalation decision making.

The *MTDR Evaluation Period* was defined as the time period from the first infusion until either: 28 days after their first infusion in Cr1 for patients who discontinued protocol therapy before Cr2, 28 days after their first infusion in Cr2 for patients who discontinued protocol therapy before Cr3, and one day prior to Cr3 for patients who received infusions in Cr3 and beyond. The MTDR was defined as the highest dose regimen with (1) at least 6 dose escalation-evaluable patients, (2) a DLT rate during the DR Escalation Evaluation Period closest to 30%, and (3) a DLT rate during the MTDR Evaluation Period of no more than of 34%. Defining the MTDR in this way ensured that the selected MTDR sustained a well-tolerated safety profile across two full courses. Subsequently, a small expansion cohort was enrolled at the MTDR with continuous safety monitoring.

## RESULTS

### Preclinical analysis of quad-CAR T cells

Quad-CAR T cells were manufactured using clinical grade lentivirus in a CGMP facility for Phase 1 studies. Optimization of transduction to achieve a balanced product defined as similar levels of integration of each CAR was done using a matrix of MOIs (Figure 1 A–D). B7-H3 CAR and IL13zetakine constructs demonstrated higher DHFRdm expression in the manufactured product, potentially reflecting higher MOI during transduction or enrichment during MTX selection (Figure 1B and Supplementary Figure 6B). Following manufacture using healthy donor human T cells, quad-CAR T cells were assessed for efficacy both in vitro and in vivo, using a model cell culture expressing all four antigens as well as GFP and firefly luciferase (U87-MG) or treatment-naive patient-derived DIPG models (PBT-22FH, PBT-24FH, PBT-27FH, and PBT-29FH). Antigen expression was assessed on U87-MG, showing 100% expression of each CAR-targeting molecule (Supplementary Figure 2A), with an expected small population of cells negative for IL-13Ra2. Cytotoxicity analysis using the IncuCyte platform shows full U87-MG tumor eradication by the quad-CAR T cell product (Supplementary Figure 2B). In an orthotopic xenograft murine tumor model, the quad-CAR T cells were able to effectively eradicate tumor to below a detectable bioluminescence signal in the majority of the animals, leading to a significant survival benefit over mock T cells alone (Supplementary Figure 2C and D). Importantly, when using four different DIPG patient-derived tumor models, the quad-CAR T cell product showed significant in vitro cytotoxicity (Supplementary Figure 2E). An in vivo orthotopic xenograft study using PBT-27FH treated with a single dose of ICV quad-CAR T cells then demonstrated significant increase in overall survival (*p* < 0.0001) (Supplementary Figure 2F). Together, preclinical data demonstrates robust activity of the quad-CAR T cells and supported the advancement to clinical trial.

### BrainChild-04: Clinical Trial Design and Patient Demographics

Based on our preclinical findings, Seattle Children’s success in delivering CAR T cells with potent antitumor activity, and evidence that repeated locoregional delivery of CAR T cells to patients with CNS tumors was feasible and well tolerated,^14–16,45,46^ we opened a phase 1 single-institution clinical trial, BrainChild-04, to accrual in May 2023.

### Patient Characteristics

Patient characteristics for the 26 patients enrolled on Arm A are shown in **Table 1**. The median time from initial diagnosis to trial enrollment among enrolled patients was 4.2 months (mean 5.8, range 2.4-17.2). Fifteen patients (57.7%) underwent a biopsy of their pontine tumor at initial diagnosis. All patients received radiation therapy before enrollment (n=26, 100%), with 3 of those patients also receiving reradiation as bridging therapy. Most of the patients (n=16, 61.5%) did not receive chemotherapy or other targeted agents prior to enrollment on trial.

### Drug Product Analysis

Flow cytometric analysis of the 21 CAR T cell drug products infused during the DLT period was performed after thaw to determine co-expression of the four constructs. All products showed high surface expression of the EGFRt transduction tag post-thaw (mean 92.5% of CD3+, range 77.6-98.7) and were majority CD8+ (mean 77.1% of CD3+, range 46.7-99.1) as shown in Supplementary Figure 6A. Within the EGFRt+ population, co-expression of the four CARs was determined via Boolean gating. Summary plots show the drug products were composed of a variety of transduction combinations, 90% of transduced cells expressed at least one CAR, and approximately half of transduced cells expressed the methotrexate-selected B7-H3 or IL-13Rα2 CARs, with mean expression of 48.0% and 53.0%, respectively; 19.5% of cells co-expressed both CARs (Supplementary Figure 6B).

### Treatment and Feasibility

A cell product approved for infusion was successfully generated for all 26 enrolled patients. Twenty-one patients received at least one CAR T cell infusion including 18 in the dose-finding cohort and 3 in the expansion cohort. Reasons for failing to receive the initial CAR T cell infusion included rapid disease progression resulting in them being ineligible to receive infusions (n=4), or patient/family preference (n=1). The median time from diagnosis to enrollment for infused patients was 3.9 months (mean 5.8, range 2.4-17.2), and the median time from enrollment to initial infusion was 1.8 months (mean 2.0, range 1.2-4.6). Three patients were removed from protocol therapy after Cr1 due to rapid disease progression. Eighteen of the infused patients initiated Cr2 and 15 of them received all intended doses in Cr2. Three patients did not receive all planned doses in Cr2. One was removed from protocol therapy after the initial infusion in Cr2 due to disease progression. Two patients missed the second infusion in Cr2: one due to neurosurgical procedure and one due to adverse event recovery, although each went on to receive one final infusion in Cr3. In DR1, 3 patients received up to 2.5x10^7^ CAR T cells/dose (DR1); in DR2, 3 patients received up to 5x10^7^ CAR T cells/dose; and in DR3, 15 patients received up to 10x10^7^ EGFRt^+^ cells/dose, identified by expression of EGFRt, which indicated co-expression of at least one and up to four of the CARs.

A total of 190 doses of quad-CAR T cells were administered with a median of 8 infusions per patient (range: 3-56), and a median of 4 courses initiated per patient (i.e., received at least one dose per course; range: 1-28). Infused patients remained on protocol therapy for a median of 3.9 months (range: 0.8-27.4).

### Safety

Possibly, probably, or definitely related adverse events (AEs) that occurred during the maximum tolerated dose regimen evaluation (MTDR) evaluation period are shown in **Table 2**. The most frequent AEs were fever (n=20, 95%), headache (n=19, 91%), nausea and/or vomiting (n=18, 86%), and fatigue (n=18, 86%). Most observed events were grade 1 or 2, and no ≥ grade 4 related toxicities occurred. Grade 3 toxicities experienced by more than one patient included headache (n=10, 47.6%), depressed level of consciousness (n=3, 14.3%), and fever (n=2, 9.5%). One patient each experienced grade 3 ataxia, back pain, dysarthria, generalized muscle weakness, unilateral muscle weakness, muscle weakness left-sided, and neck pain. No DLTs occurred throughout the entire MTDR evaluation period and DR3 (the highest DR) was selected as the MTDR.

Intratumoral hemorrhage resulted in a DLT in our single antigen CAR T cell trial.^16^ and thus was analyzed. No intratumoral hemorrhage was observed on BrainChild-04 Arm A. Typically, patients experienced the highest grade of each AE during their first two courses. Few patients experienced a new grade 3 AE after Cr 2, including grade 3 blurred vision (n=1), headache (n=1), acoustic nerve disorder NOS (n=1), and eye pain (n=1). One patient experienced a DLT (grade 3 acoustic nerve disorder, possibly related to investigational product) after the first infusion in Cr3, but per protocol, this did not contribute to MTDR decision making.

All patients who experienced headaches received IV magnesium sulfate, 9 patients (47.4% of all patients who experienced headaches thought to be related to CAR T cells) received oral opioids, and 11 patients (57.9%) received IV opioids. Dexamethasone was administered for grade 3 headaches or transient neurologic changes in 6 patients. Bevacizumab was given in addition to dexamethasone to two patients for transient neurologic changes. Anakinra was administered in one patient for grade 3 headache and altered level of consciousness that were initially deemed possibly related to CAR T cells (although it was subsequently attributed to disease progression following neuroimaging) but did not lead to clinical improvement. No other immunomodulatory agents were administered.

### Tumor Inflammation Associated Neurotoxicity

TIAN^28,29^ was retrospectively identified in 17 of 21 patients (81%) treated on Arm A of this trial. The highest observed grade of TIAN was grade 3 and the observed TIAN grade peaked at Cr1 Week (W)3 in all patients and persisted at maximum recorded grade through Cr2 and 3 in 8 of 17 patients (47%). TIAN most commonly presented with transient headaches with fever (n=17), other pain with fever (n=2), paresthesias (n=2), and worsening lower extremity weakness (n=2).

### Clinical Outcomes

Of the 21 evaluable patients, one achieved a partial response and 15 had stable disease (Supplementary Figure 4). Five patients whose best overall response was stable disease experienced clinical improvement following CAR T treatment characterized as improvement or resolution in tumor-related neurologic deficits on neurologic exam. This included an average reported improvement in performance status of 20 points (range 10-30) within patients with stable disease. The median survival from initial CAR T infusion was 8.6 months (95% CI: 5.2-10.3) (Figure 3). All deaths were due to disease progression. For infused patients who had not progressed before their initial infusion (n=15), median survival from initial infusion was 9.6 months (95% CI: 4.2-14.1), compared to 7.4 months (95% CI: 2.0-NR) for those who had progressed by the time of initial infusion (n=6). Median survival from diagnosis for infused patients was 18.5 months (95% CI: 13.1-23.9). At the time of analysis, 6 patients are still alive. One patient continues on protocol therapy and has received a total of 66 infusions.

### CAR T Cell Detection

CAR T cell detection in the CSF of patients is depicted in Figure 4. From Cr1 the initiation of Cr 2 (Cr2.W1.Pre), CAR T cells were detected via flow cytometry in 51 out of 77 (66.2%) analyzed CSF samples. Comparing the one matched pre/post CAR infusion timepoints at Cr1.W2, the median CAR T cell detection at pre-infusion (Cr1.W2.Pre) was 0.4% of lymphocytes for the lower dose patients (DR1 and DR2 combined, N = 6) and 0.6% for the higher dose patients (DR3 including expansion cohort, N = 15). Following the CAR infusion (Cr1.W2.Post), median CAR T cell engraftment was 2.4 % for patients receiving the higher dose regimen compared to 0.6% within the lower dose regimen. Out of 117 peripheral blood samples analyzed during the same interval, CAR T cells were detected in two samples (S025 Cr1.W4 and S011 Cr1.W2.Pre).

While the flow panel used for CAR T cell detection included markers associated with T cell activation, the limited number of cells in the CSF inhibited the ability to perform robust evaluation. A limited analysis of CD8+ cells was performed by first screening for samples with lymphocyte numbers above the limit of quantitation (LoQ) of 200 cells for the assay (Supplementary Figure 5A) at each collection timepoint. Next, the number of CD8+ and CD4+ in the EGFRt+ CAR T cell population was evaluated to determine whether sufficient cells were collected to allow for further analysis. As the analysis of interest would result in dividing each population into 16 subpopulations based on co-expression of the four activation markers included in the panel, a minimum threshold of 50 cells was set to ensure sufficient event distribution across the Boolean gates (Supplementary Figure 5B). Only 10 samples across two collection timepoints contained CD4+ EGFRt+ numbers above this threshold, while 28 samples with CD8+ EGFRt+ were above threshold and determined eligible for further evaluation. A comparison of activation marker expression in these CD8+ EGFRt+ cells was performed against CD8+ EGFRt− cells collected at the same timepoints. We observed that EGFRt− cells were predominately negative for all markers, including limited expression of PD-1+ alone. Conversely, EGFRt+ cells display a variety of activation marker expression including TIM-3+ alone, LAG-3+TIM-3+ dual positive, and LAG-3+PD-1+TIM-3+ triple positive, highlighting the heterogenous phenotypic states of the CAR positive cells (Supplementary Figure 5C). The expression of 41bb was also assessed, and minimal 41bb expression was detected, either alone or in combination with any of the other associated markers.

### CSF Cytokine Analysis

Concentrations of 47 cytokines were analyzed during Cr1 and 2 for those patients in the dose finding cohorts (15 patients). Differential analysis using estimated marginal means (EMM) of matched pre- and post-infusion samples at W2of Cr1, Cr1.W2. Pre and Cr1.W2.Post, revealed a significant elevation of key inflammatory markers. Notably, CXC motif chemokine ligand 10 (CXCL10), interferon-gamma (IFNy), and serum amyloid A (SAA) exhibited a greater-than-4-fold increase. Furthermore, 1-to-4-fold elevations in interleukin-6 (IL-6), interleukin-8 (IL-8), interleukin-1 receptor antagonist (IL-1RA), intercellular adhesion molecule 1 (ICAM-1), thymus and activation-regulated chemokine (TARC), macrophage-derived chemokine (MDC), and C-reactive protein (CRP) suggest robust T cell activation and an inflammatory immune response within the CSF (Figure 5A). To evaluate longitudinal trends, baseline levels across subsequent courses were examined in a subset of patients who completed at least four courses (n = 8; S011, S014, S017, S021, S025, S034, S036, S039) (Figure 5B). At the start of Cr2 (Cr2.W1.Pre), all patients demonstrated elevated baseline levels of TARC, IL-12/IL-23p40, and IFNy compared to Cr1 (Cr1.W1.Pre). Similar trends for CXCL10 were observed in seven out of eight patients. In later courses, CRP and SAA displayed heterogenous trends. Collectively, these shifts in cytokine profiles, both in direct response to a single CAR T cell infusion and longitudinally during CAR T therapy, reflect a dynamic and evolving tumor immune microenvironment (TIME).

### Targeted Proteomics analysis

Quantitative targeted proteomics (i.e., Parallel Reaction Monitoring, PRM) analysis was conducted using a multiplexed panel targeting 117 proteins (144 peptides) related to immuno-oncology.^40,42^ A total of 99 CSF specimens across 15 patients were analyzed from which a total of 47 proteins (51 peptides) were detected in the CSF in over half of the specimens. An overview of quality control is shown in Supplementary Figure 7 and data tables are provided in **Supplementary Table 3**.

Differential protein expression analysis was conducted to evaluate treatment-associated changes in CSF protein profiles across longitudinal timepoints relative to baseline. A total of 18 proteins demonstrated significant increases (adjusted p < 0.1) following treatment at both Cr1.W2.Pre and Cr1.W2.Post, with sustained elevation persisting through Cr1.W3 (Figure 6A–C). Enriched proteins are associated with T-cell infiltration and T-cell receptor (TCR)/CAR signaling, cytotoxic effector function, interferon-gamma (IFN-γ)/Janus kinase-signal transducer and activator of transcription (JAK-STAT) signaling, myeloid and innate immune activation, early checkpoint and exhaustion programs, and anti-inflammatory counter-regulation. Notably, CXCL9 and IDO1 showed sustained induction across these early post-treatment timepoints (Figure 6E–F). An additional 44 proteins demonstrated significant increases at Cr1.W2.Post that remained elevated at Cr1.W3 (Figure 6B–C). These proteins are associated with antigen presentation, helper T cell co-stimulation, B cell and tertiary lymphoid structure signaling, myeloid reprogramming, blood-brain barrier and endothelial activation, cytokine signaling, sustained checkpoint and exhaustion pathways, adenosine-mediated immunosuppression, and apoptosis. In contrast, 30 proteins exhibited a transient increase restricted to Cr1.W2.Post without sustained elevation at Cr1.W3 (Figure 6B). These transiently induced proteins are primarily associated with proliferation, DNA damage checkpoint and repair, and metabolic signaling pathways.

Longitudinal trend analysis was subsequently performed using a linear mixed-effects model incorporating pre-treatment samples from Cr1.W1, Cr1.W2, Cr1.W3, Cr2.W1, Cr3.W1, Cr4.W1, Cr5.W1, and Cr7.W1, with timepoints modeled as ordinal variables. Twelve proteins demonstrated a sustained positive longitudinal trend across treatment courses (Figure 6D). These proteins represented a subset of those elevated at Cr1.W2.Post and sustained through Cr1.W3 and were enriched for IFN-γ-driven inflammatory tumor microenvironment signaling, B cell follicular chemokine activity, checkpoint signaling, and DNA damage-associated pathways. Among these, CXCL13 exhibited the strongest positive longitudinal trend (Figure 6G).

Although no proteins demonstrated a significant negative longitudinal trend following FDR correction, several proteins showed nominal decreases over time. Among these, MSH3, a mismatch repair-associated protein, demonstrated a marginally significant decreasing trend (p < 0.05) beginning after Cr2.W1 (Figure 6H), potentially reflecting early mismatch repair weakening or hypermutator evolution under sustained immune pressure. One limitation of this trend analysis is that patients who received more treatment cycles, for example those with fewer toxicity-related effects, contributed more data points, which could bias the results. In particular, the apparent decreasing trend in MSH3 may be driven by a small number of patients who received more than four treatment cycles. Therefore, these results should be interpreted with caution.

## DISCUSSION

The BrainChild-04 first-in-human phase 1 trial met its primary objectives related to Arm A, demonstrating feasibility of manufacturing and administering a locoregional quad-CAR T cell product to children and young adults with DIPG or pontine DMG while maintaining an acceptable safety profile across dose levels. This study represents the first clinical application of a quadruple antigen-targeting CAR T cell platform simultaneously targeting the B7-H3, EGFR806, HER2, and IL-13Rα2 tumor-associated antigens. Successful manufacturing of this complex autologous product using four separate lentiviral constructs, with reproducible generation of all four antigen-specific CAR T cell populations and consistent product quality across patients, demonstrates the technical feasibility and scalability of this multi-antigen approach. Although this study was not designed to determine whether the relative composition of individual CAR T cell populations influences expansion, persistence, or clinical activity, these product characteristics provide an important foundation for optimizing future multi-antigen CAR T cell therapies. Repeated ICV administration was feasible without lymphodepletion, allowing patients to receive multiple treatment courses and supporting the practicality of sustained locoregional immune engagement in CNS tumors.

Lymphodepleting chemotherapy was intentionally omitted from this platform. While lymphodepletion has been shown to enhance CAR T cell expansion and persistence in hematologic malignancies through elimination of endogenous lymphocytes and increased availability of homeostatic cytokines,^47^ its role in locoregionally delivered CAR T cell therapy for CNS tumors remains uncertain. The demonstration of serial CAR T cell detection within the CSF and sustained modulation of the CNS immune microenvironment^32,44^ suggests that meaningful local immune activation may be achieved in the absence of systemic lymphodepletion. Nevertheless, whether incorporation of a pretreatment conditioning regimen, most likely targeted against the tumoral myeloid population rather than the systemic lymphoid population, may augment efficacy and/or tolerability remains an important unanswered question that warrants further preclinical and clinical evaluation. Future studies should prospectively evaluate whether conditioning regimens to enhance CAR T cell expansion and persistence can improve anti-tumor efficacy in this setting, while balancing potential efficacy gains against the favorable safety profile and logistical advantages of repeated ICV dosing without lymphodepletion.

These findings build upon prior experience with locoregional CAR T cell therapy in pediatric CNS malignancies while extending the field toward strategies designed to overcome tumor heterogeneity and antigen escape. In particular, the BrainChild-03 experience with B7-H3-directed CAR T cells provides an important comparator for the current study. In the DIPG-specific Arm C, 21 patients received repeated ICV B7-H3 CAR T cell therapy, with 253 total doses administered and a median of 9 doses per patient.^16,^ The highest planned dose regimen, escalating to 1×10^8^ cells per dose, was established as the maximally tolerated dose regimen, with headache, fatigue, and fever among the most common adverse events and one dose-limiting toxicity of intratumoral hemorrhage. More recently, BrainChild-03 Arm B demonstrated this approach for non-pontine DMG and other recurrent or refractory pediatric CNS tumors.^45^ Among 36 enrolled patients, 8 had DMG and 26 patients overall received therapy, with 181 ICV doses administered and a median of 7 doses per patient. The same nominal cell dose of 1×10^8^ CAR T cells per dose was identified as the maximally tolerated dose regimen, with no dose-limiting toxicities observed. Headache, fever, and nausea were the most common adverse events, and two patients achieved partial responses. Median survival from first infusion was 11.5 months across the overall heterogeneous cohort; because DMG-specific survival and response outcomes were not separately reported, these findings should not be interpreted as a DMG-specific efficacy estimate.^42^ Together, these studies establish the feasibility of repeated ICV B7-H3 CAR T cell administration across both pontine and non-pontine DMG populations and provide a clinical foundation for evaluating multi-antigen and combinatorial approaches.

Compared with these prior single-antigen CAR T cell studies, the inflammatory signal observed in the current trial appeared more pronounced, with increased constitutional and neurologic toxicities including fever, headache, and fatigue, although the majority of events remained grade 1-2. Prior studies of GD2-directed CAR T cells demonstrated encouraging biologic and clinical activity but identified tumor inflammation-associated neurotoxicity as a toxicity concern.^48^ Similarly, B7-H3-directed CAR T cells in BrainChild-03,^16,45^ and IL13Rα2-directed CAR T cell therapy in HGG^19,34^ demonstrated the feasibility of locoregional delivery with manageable inflammatory toxicities, while HER2- and EGFR-targeted approaches, including BrainChild-02 in patients with a variety of high-grade CNS tumors,^14,18^ generally reported lower rates of neurotoxicity. The comparatively heightened inflammatory profile observed with quad-CAR T cells may reflect enhanced immune activation associated with simultaneous targeting of four tumor-associated antigens. However, cross-trial comparisons of toxicity should be interpreted cautiously given differences in patient populations, disease burden, treatment schedules, CAR constructs, and toxicity ascertainment. Overall, these findings suggest that increasing antigen breadth may enhance immune engagement within the CNS but may also increase the intensity of treatment-associated inflammation, underscoring the importance of careful toxicity monitoring and optimization of multi-antigen CAR T cell design.

Importantly, correlative studies supported biologic activity within the CNS, demonstrating CAR T cell detection in CSF across sequential treatment courses and dynamic modulation of the CNS immune microenvironment. Proteomic analyses identified induction of pathways associated with T cell trafficking, cytotoxic effector function, IFN-γ signaling, innate immune activation, antigen presentation, and adaptive immune organization.^41,49^ Sustained increases in CXCL9, CXCL13, and IDO1 suggest ongoing immune activation accompanied by compensatory immunoregulatory signaling, findings consistent with prior studies of CAR T cell therapy and anti-tumor immune responses in CNS malignancies and other solid tumors.^19,48,50–52^ Together, these data support the hypothesis that repeated locoregional CAR T cell administration may progressively remodel the CNS tumor microenvironment and promote localized adaptive immune responses over time.

Several limitations should be acknowledged. As a phase 1 study, the trial was not designed or powered to definitively evaluate efficacy, and survival outcomes should therefore be interpreted cautiously. The cohort was relatively small and somewhat heterogeneous, including patients treated both before and after disease progression, which complicates interpretation of clinical outcomes. Importantly, no patients in this cohort received re-irradiation during the study, providing a relatively uniform treatment context and distinguishing other cohorts of patients with DIPG/DMG treated with CAR T cells in which re-irradiation was incorporated for some patients.^48^ In addition, the absence of mandatory tumor antigen confirmation at enrollment limits the ability to correlate antigen expression with response or toxicity. While the correlative analyses provide important biologic insight, these findings remain exploratory and hypothesis-generating. Larger studies with more homogeneous patient populations and integrated longitudinal biomarker analyses will be required to better define predictors of response, resistance, and treatment-related neurotoxicity.

Despite these limitations, several findings from this study inform the future development of CAR T cell therapies for CNS tumors. First, the reproducible manufacture and repeated administration of a complex, multi-antigen CAR T cell product establishes the feasibility of advancing combinatorial targeting strategies into later-phase studies. Although this trial relied on the availability of four previously manufactured GMP vector lots, ongoing efforts to streamline the platform toward a single vector incorporating compatible tandem scFv combinations may facilitate broader clinical translation. Beyond manufacturing feasibility, the detection of CAR T cells and evidence of sustained immune activation in the CSF suggest that repeated locoregional dosing may support persistent CAR T cell presence and ongoing immune engagement within the CNS. However, the concurrent emergence of checkpoint, exhaustion, and adenosine-mediated immunosuppressive pathways points to potential mechanisms of adaptive resistance that could inform future combination strategies. These findings also underscore the value of longitudinal CSF proteomic and cytokine profiling as a minimally invasive approach to characterizing evolving immune responses during therapy. Incorporating these biomarkers into future trials may help refine patient selection, optimize dosing schedules, and inform toxicity mitigation strategies, including cytokine-directed interventions to improve the therapeutic window.

In summary, this study demonstrates that the manufacture and subsequent repetitive ICV administration of autologous quad-targeting CAR T cells is feasible and clinically tolerable in children and young adults with DIPG or pontine DMG. Multi-antigen targeting produced evidence of biologic activity within the CNS, including CAR T cell detection and dynamic immune remodeling, while highlighting the challenges associated with treatment-related inflammation. Collectively, these findings provide an important foundation for future studies to evaluate whether multi-antigen locoregional CAR T cell therapy can improve the durability and efficacy of immunotherapy for these highly aggressive and currently incurable pediatric CNS tumors.

## Supplementary Material

1

## Figures and Tables

**Figure 1:**
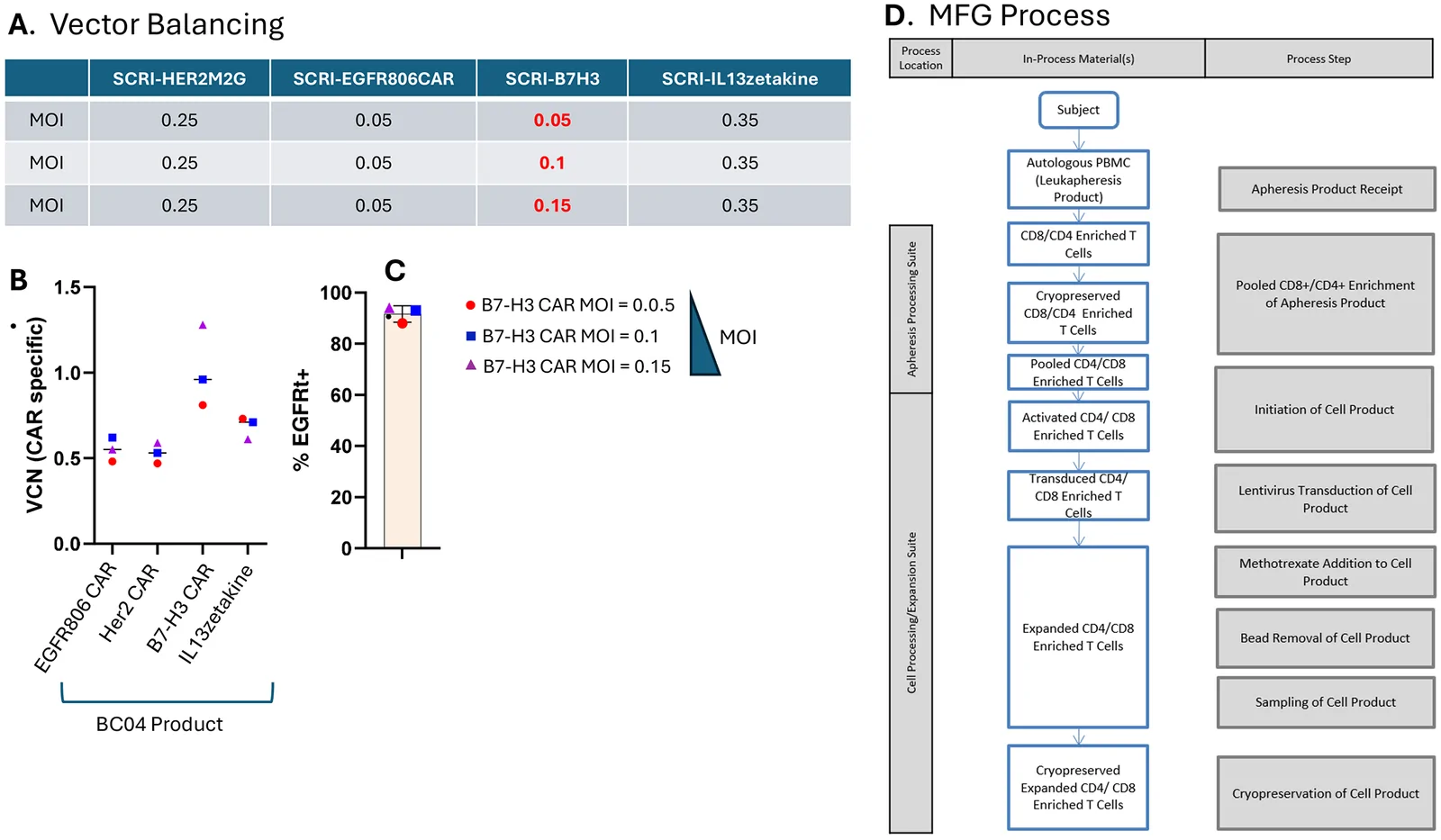
Optimization and validation of vector MOI for full-scale GMP BC-04 CAR T-cell manufacturing platform

**Figure 2:**
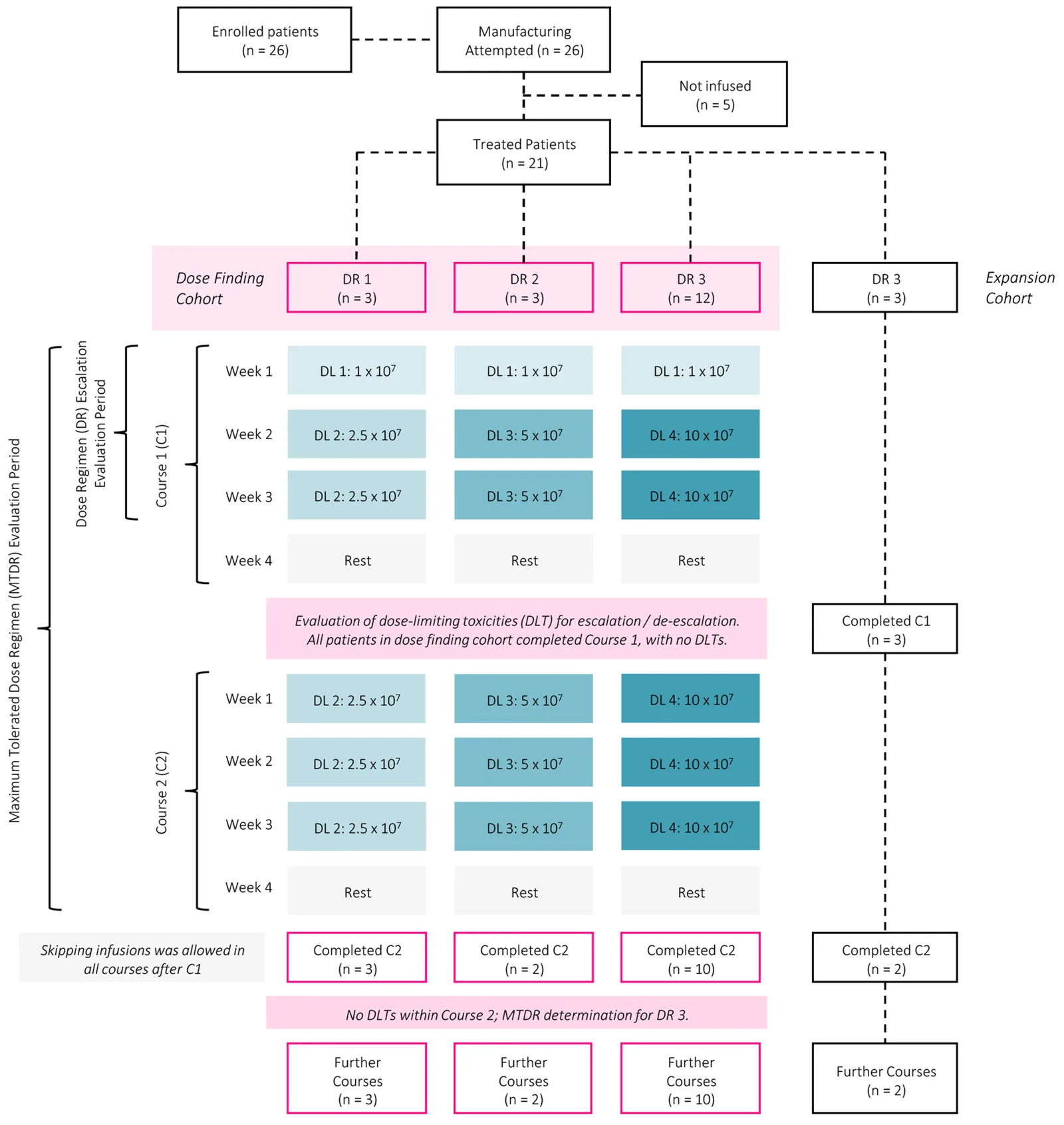
CONSORT diagram of subjects enrolled on BrainChild04 Arm A

**Figure 3A-C:**
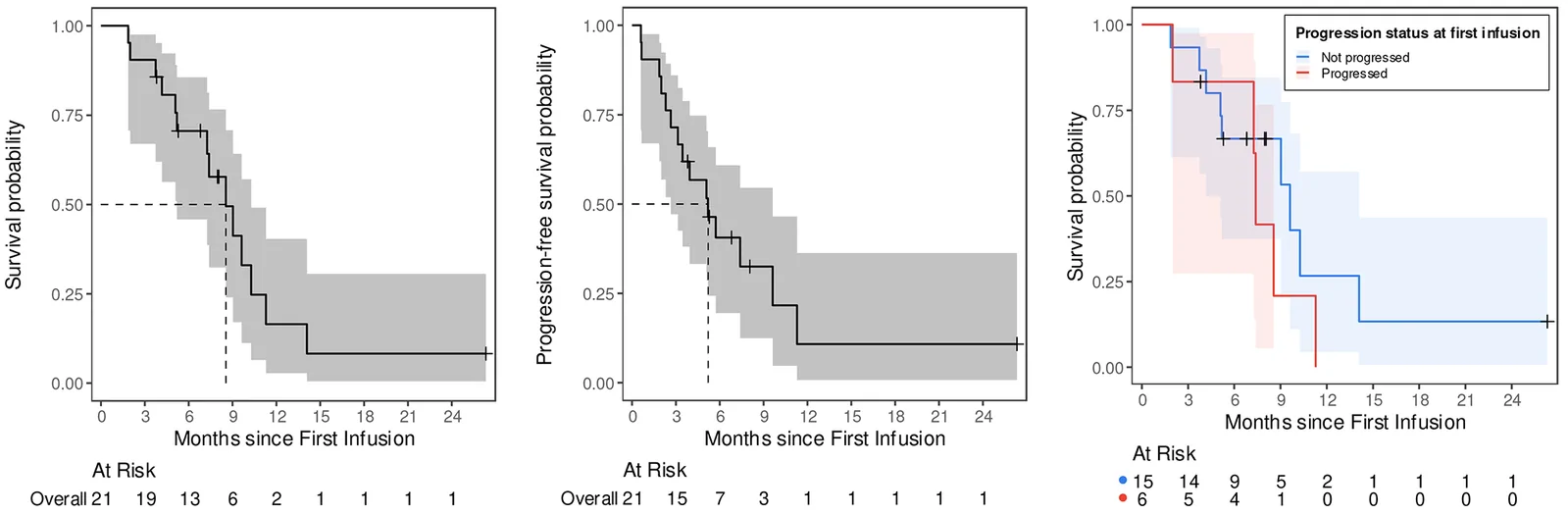
Kaplan Meier Curves: overall survival (OS) from CAR-T infusion, progression-free survival (PFS) from CAR-T infusion, and overall survival (OS) stratified by progression status at first infusion

**Figure 4:**
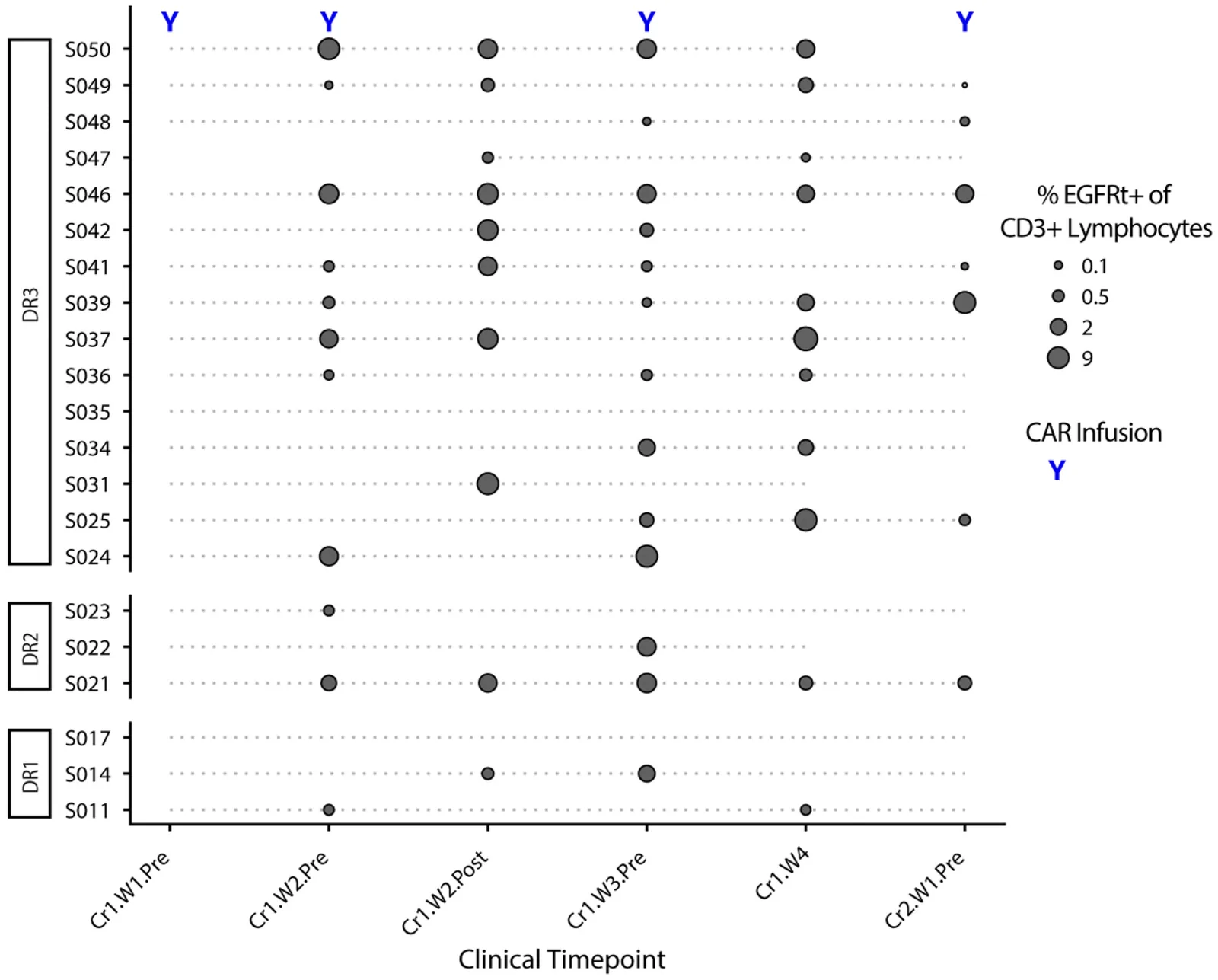
Detection of CAR T cells in CSF via flow cytometry

**Figure 5:**
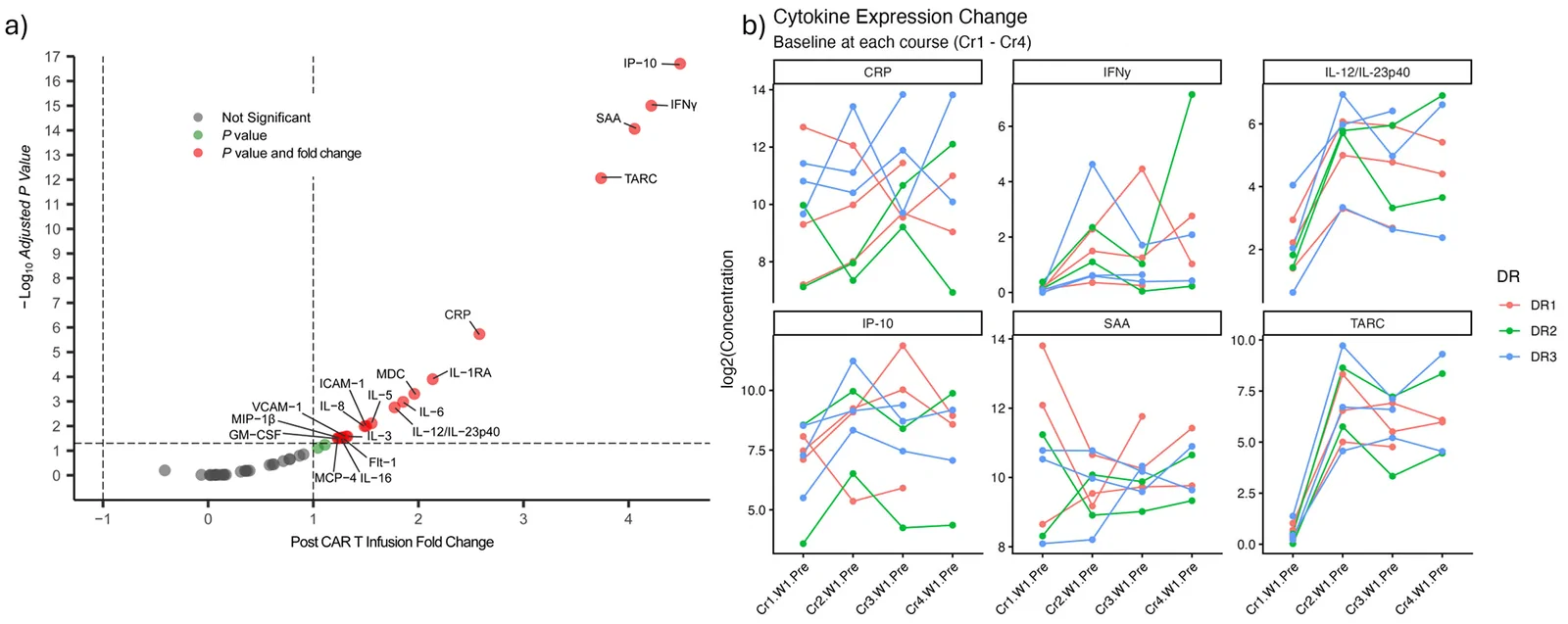
Fold change in CSF Cytokines and Chemokines following CAR T Infusion during Initial Courses

**Figure 6:**
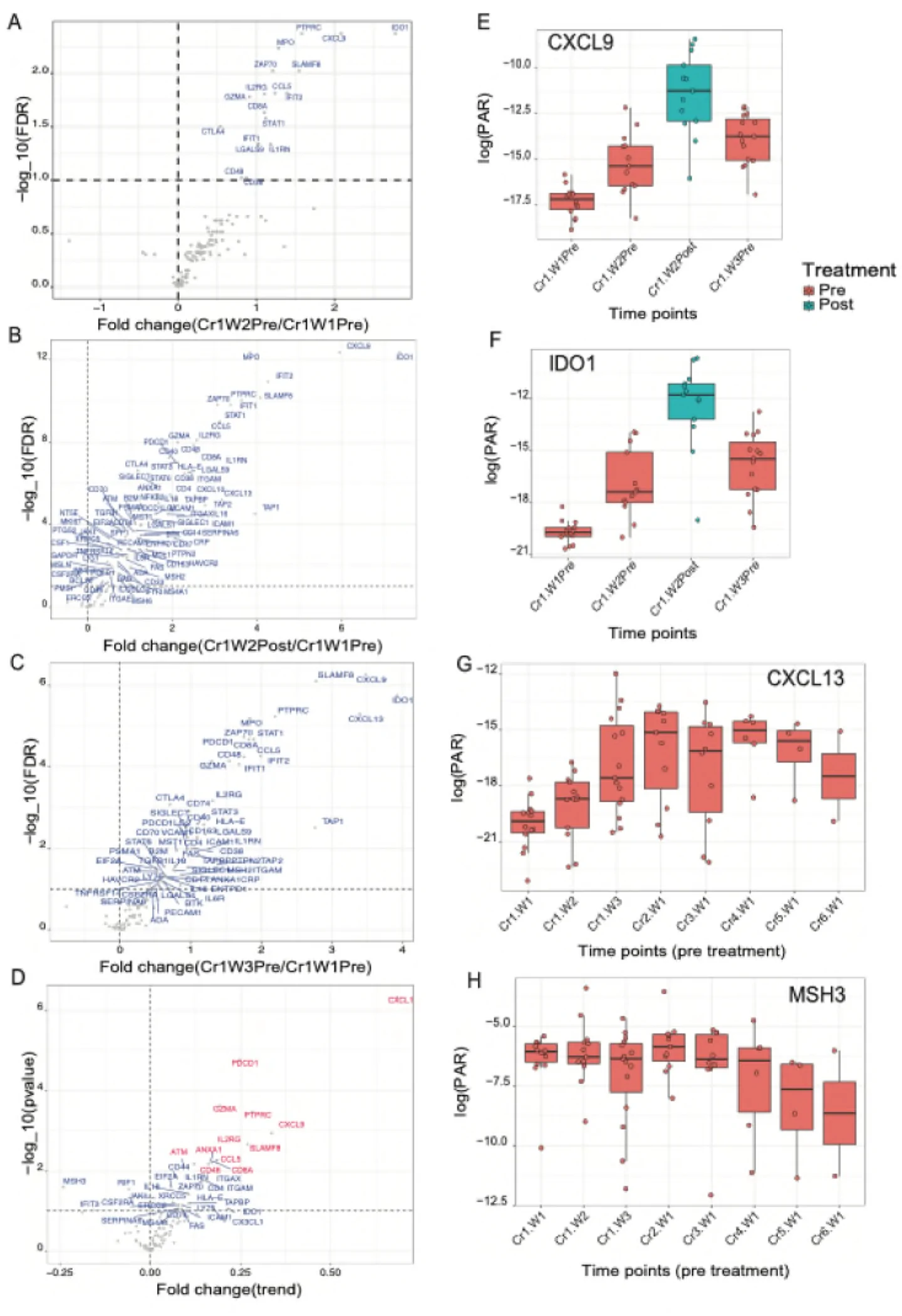
Changes in proteomic expressions after treatment and sustained effect from baseline

## Data Availability

All requests for raw and analyzed data and materials will be promptly reviewed by the intellectual property office of Seattle Children’s Research Institute to verify if the request is subject to any intellectual property or confidentiality obligations. Raw preclinical and clinical data is stored at Seattle Children’s with indefinite appropriate backup. Patient-related data not included in the paper were generated as part of clinical trials and may be subject to patient confidentiality. Any data and materials that can be shared will be released via a Material Transfer Agreement.
