# Supplementary material for: First-in-human quad-targeting CAR T cells for diffuse intrinsic pontine glioma: a phase 1 trial
Source: Res Sq. 2026 Oct 27:rs.3.rs-11447687. Preprint. [Version 1] doi: 10.21203/rs.3.rs-11447687/v1 (PMC13734270; doi:10.21203/rs.3.rs-11447687/v1)
Supplement: Supplementary file 1 [file NIHPPrs11447687v1-supplement-1.pdf]

Tables 1 and 2 are available in the supplementary files section

TABLES AND FIGURES

Tables

Table 1: Patient characteristics of study participants enrolled in BrainChild-04 Arm A.

Table 2: Comprehensive AE list during the first two courses (possibly, probably, definitely related to investigational product)

Supplementary Figures

Supplementary Figure 1: Representative Flow Gating

Supplementary Figure 2: Preclinical analysis of Quad-CAR T product

Supplementary Figure 3: Common Adverse events by course

Supplementary Figure 4: Changes on MRI pre and post CAR T infusion

Supplementary Figure 5: CSF Activation marker detection

Supplementary Figure 6: Drug Product Characterization

Supplementary Figure 7: Overview of targeted proteomics quality control results

Supplementary Tables:

Supplementary Table 1: Regents used for CAR detection by flow cytometry in CSF

Supplementary Table 2: Regents used for CAR detection by flow cytometry in Drug Products

Supplementary Table 3: Proteomics data set

Supplementary Table 4: Evaluation of overall response

Supplementary Figures

Supplementary Figure 1: Representative Flow Gating

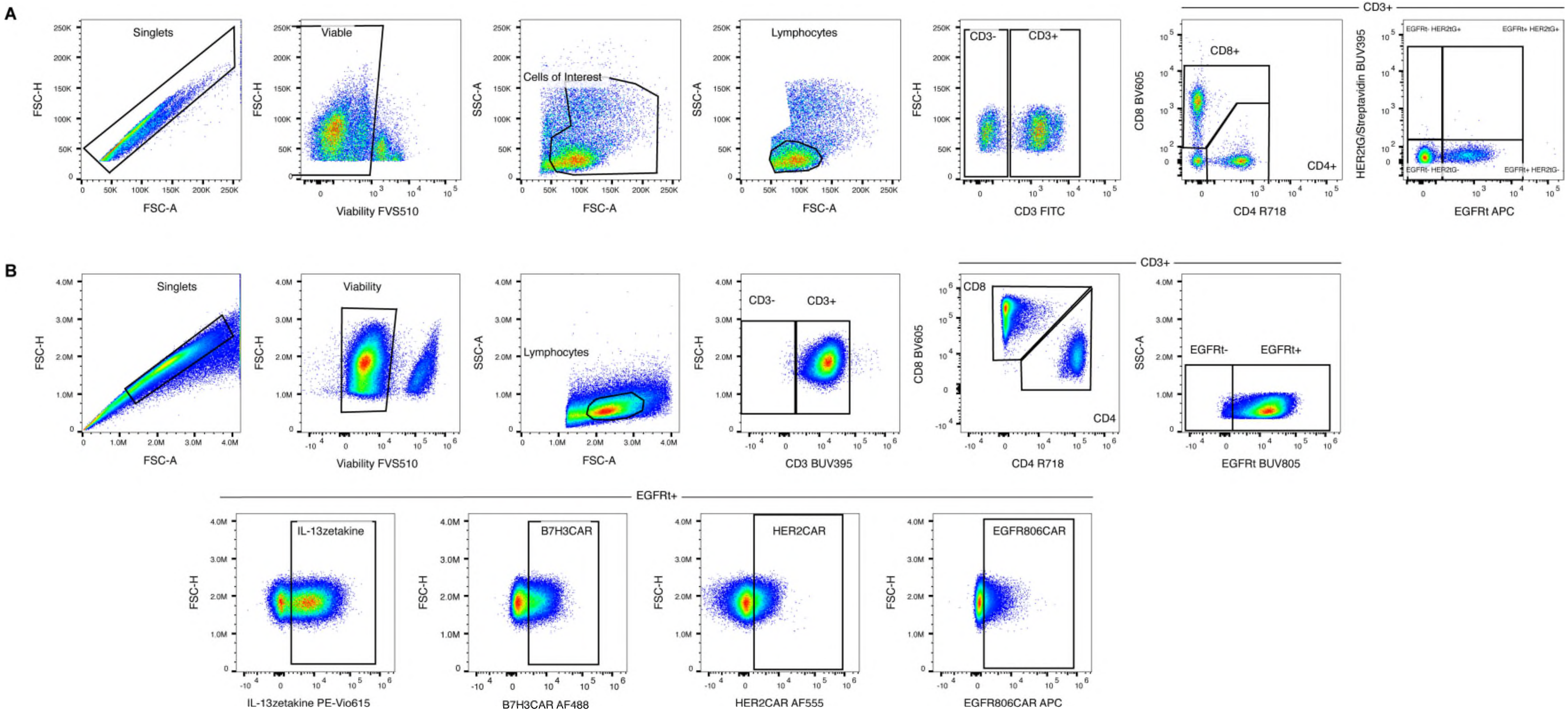

Supplementary Figure 2: Preclinical Analysis of Quad CAR T Product

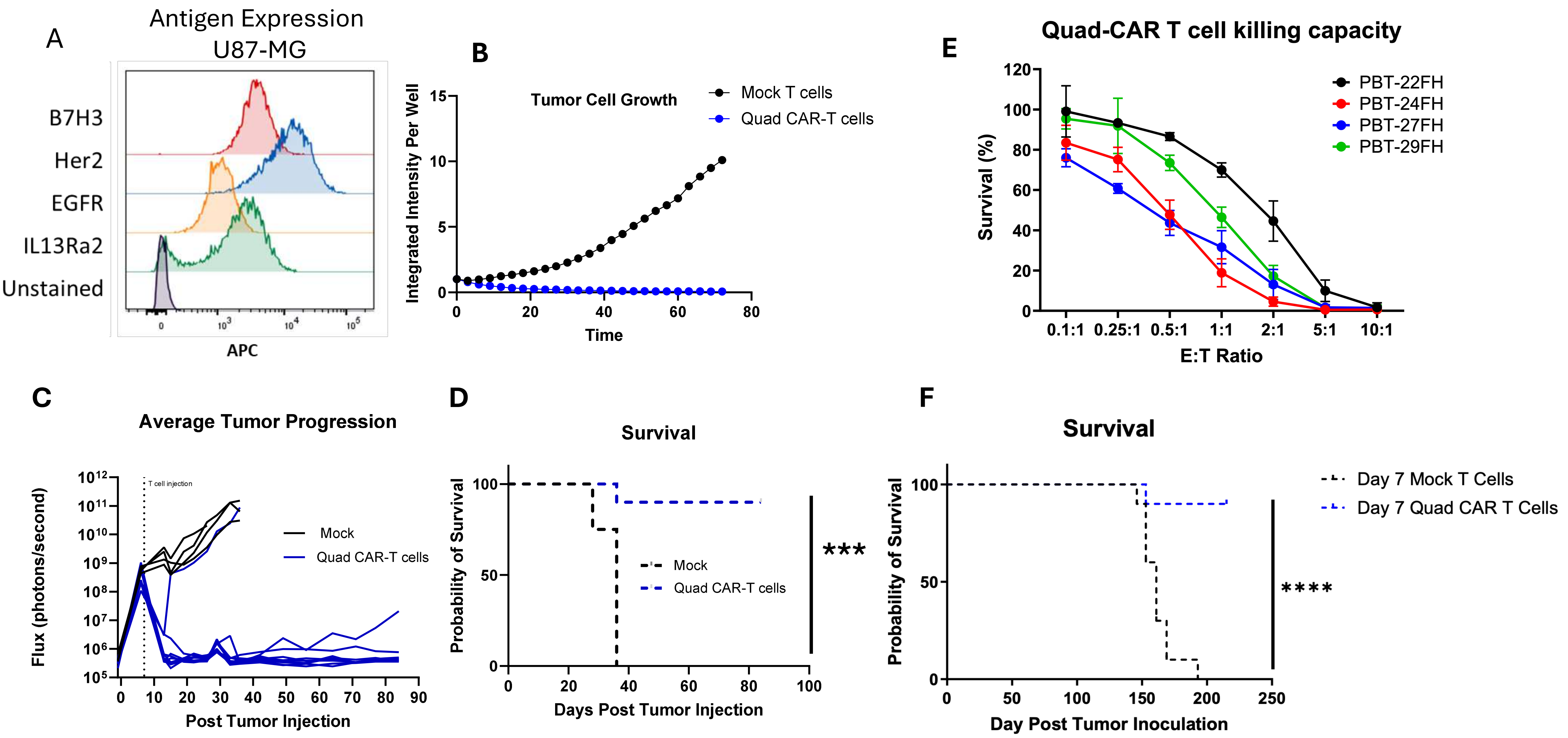

Supplementary Figure 3: Common adverse events by course

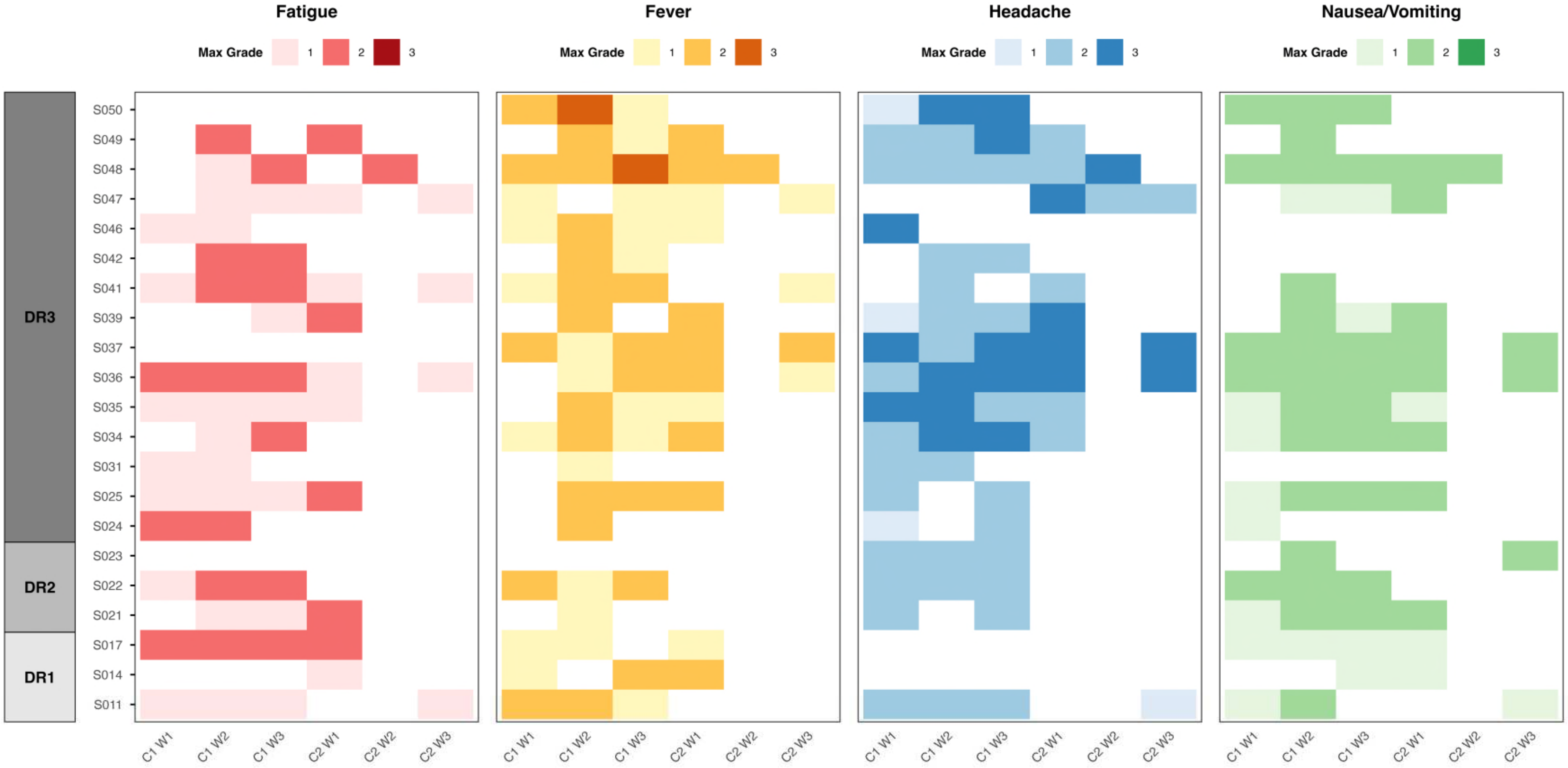

Supplementary Figure 4 A and B: Changes on MRI pre and post CAR T infusion

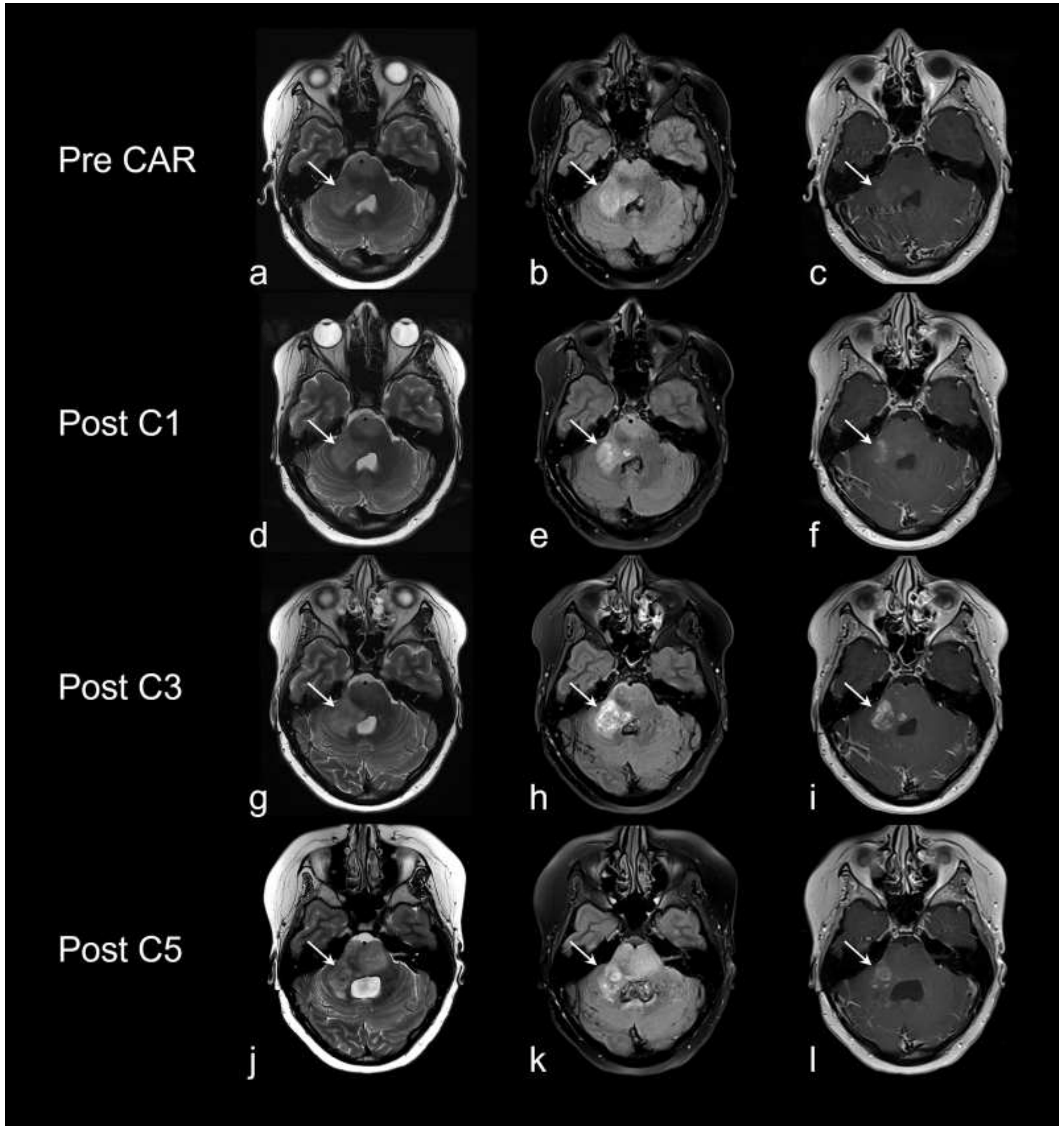

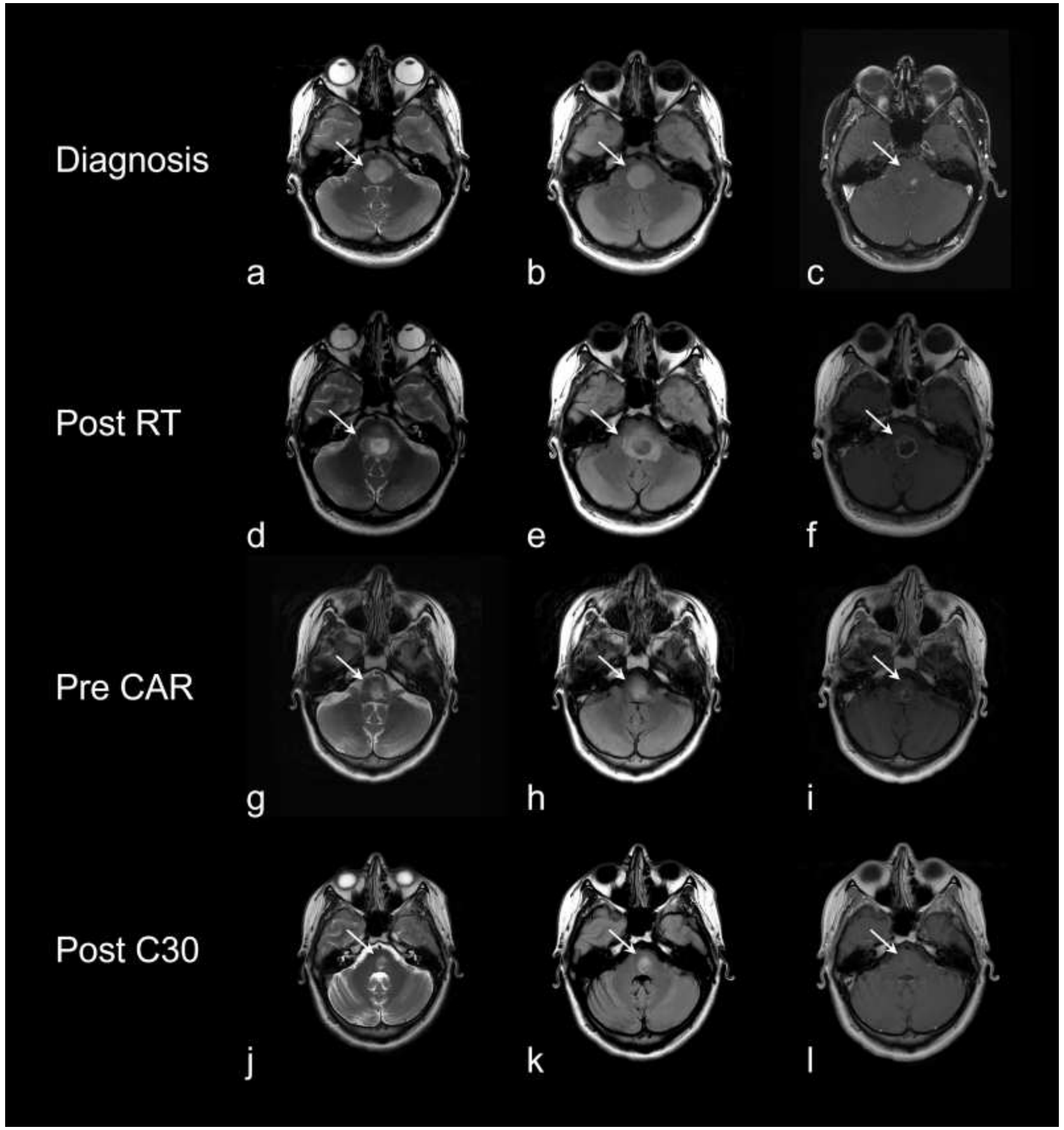

Supplementary Figure 5: CSF activation marker detection

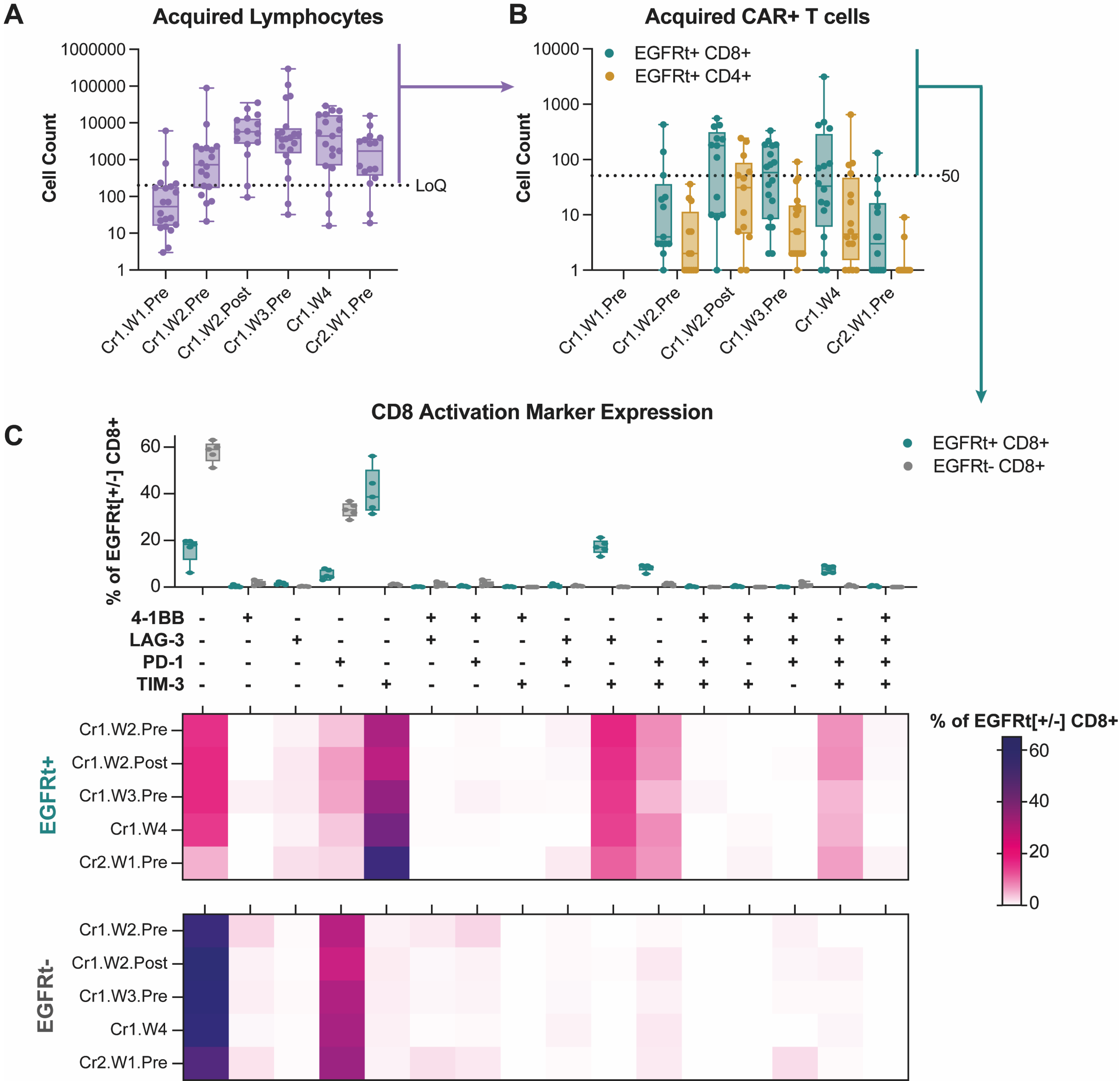

Supplementary Figure 6: Drug product characterization

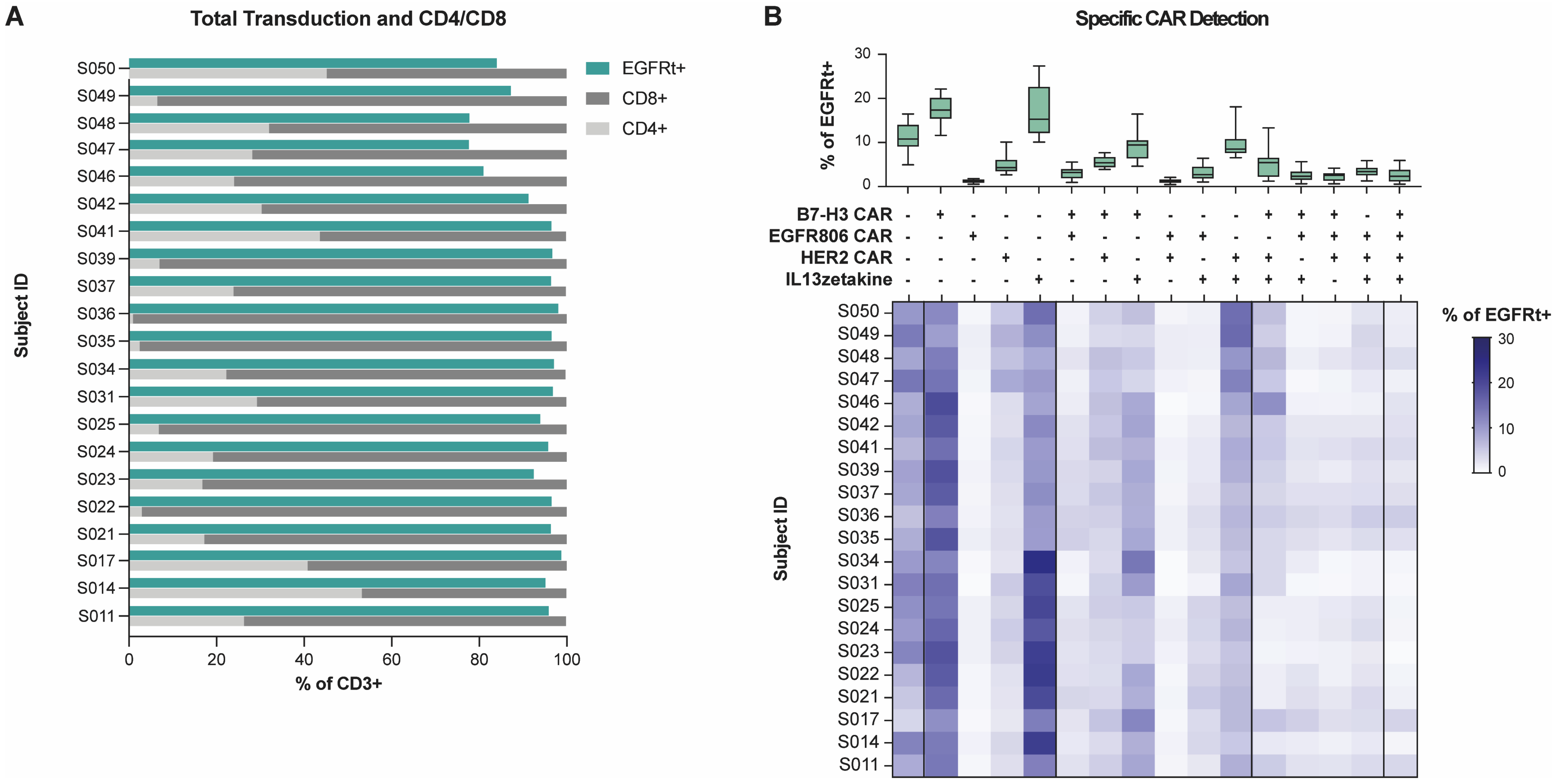

Supplementary Figure 7: Overview of targeted proteomics quality control results

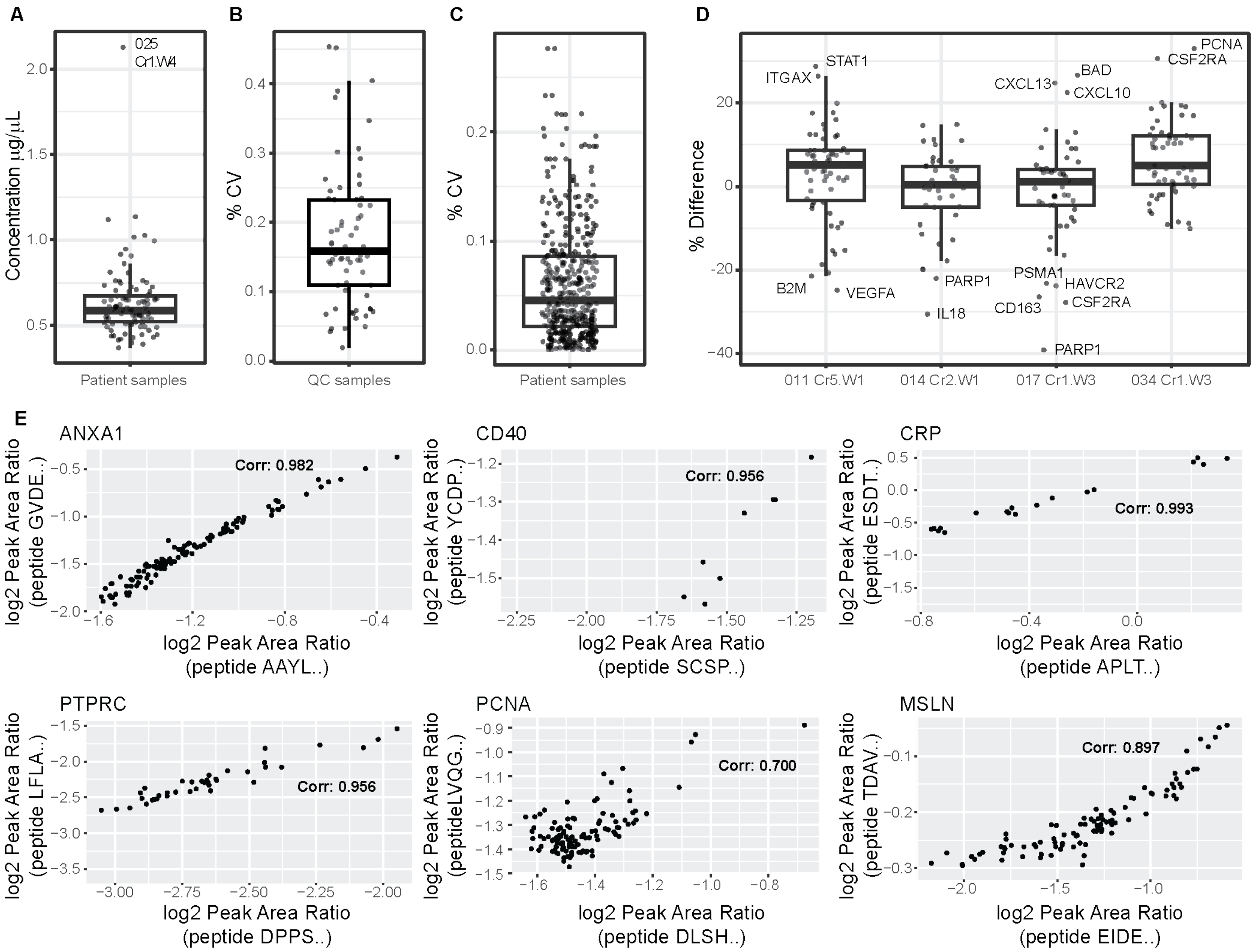

Supplementary Table 1: List of CAR constructs

CAR	Hinge	Transmembrane domain	Co-stim	Transduction tag	Drug resistance
B7-H3	IgG4:CH3	CD28	41bb:CD3zeta	EGFRt	MTX (DHFRdm)
IL13zetakine	IgG4:CH3	CD28	41bb:CD3zeta	EGFRt	MTX (DHFRdm)
EGFR806	IgG4	CD28	41bb:CD3zeta	EGFRt	none
Her2	IgG4:CH3	CD28	41bb:CD3zeta	EGFRt	none

Supplementary Files

This is a list of supplementary files associated with this preprint. Click to download.

- SupplementaryTable4OverallResponse.docx
- BC04ArmATablesSeptember2026.docx
- SupplementaryTables1and2September2026.docx
- SupplementaryTableProteomicscopy.xlsx
- NatureMedicine.pdf
- CompletewithDocusignBrainChild04ProtocolSIGNED.pdf
